# Oncogenic Proteomics Approaches for Translational Research and HIV-Associated Malignancy Mechanisms

**DOI:** 10.3390/proteomes11030022

**Published:** 2023-07-04

**Authors:** Eduardo Alvarez-Rivera, Emanuel J. Ortiz-Hernández, Elyette Lugo, Lorraine M. Lozada-Reyes, Nawal M. Boukli

**Affiliations:** 1 Biomedical Proteomics Facility, Department of Microbiology and Immunology, Universidad Central del Caribe, School of Medicine, Bayamón, PR 00960, USA; 2 Department of Biology, Universidad de Puerto Rico, Bayamón, PR 00960, USA

**Keywords:** quantitative proteomics, mass spectrometry, biomarkers, cancer, co-morbidity, HIV-associated malignancies

## Abstract

Recent advances in the field of proteomics have allowed extensive insights into the molecular regulations of the cell proteome. Specifically, this allows researchers to dissect a multitude of signaling arrays while targeting for the discovery of novel protein signatures. These approaches based on data mining are becoming increasingly powerful for identifying both potential disease mechanisms as well as indicators for disease progression and overall survival predictive and prognostic molecular markers for cancer. Furthermore, mass spectrometry (MS) integrations satisfy the ongoing demand for in-depth biomarker validation. For the purpose of this review, we will highlight the current developments based on MS sensitivity, to place quantitative proteomics into clinical settings and provide a perspective to integrate proteomics data for future applications in cancer precision medicine. We will also discuss malignancies associated with oncogenic viruses such as Acquire Immunodeficiency Syndrome (AIDS) and suggest novel mechanisms behind this phenomenon. Human Immunodeficiency Virus type-1 (HIV-1) proteins are known to be oncogenic per se, to induce oxidative and endoplasmic reticulum stresses, and to be released from the infected or expressing cells. HIV-1 proteins can act alone or in collaboration with other known oncoproteins, which cause the bulk of malignancies in people living with HIV-1 on ART.

## 1. Introduction

The introduction of mass spectrometry (MS) has become more popular in recent years, making it an attractive approach to understanding the complexity of cancer-mediated signaling; as such, this has allowed the assessment of individual unique biosignature profiles [1,2]. Specifically, this platform provides insight into mechanistic processes such as protein interaction, structure, function, and post-translational modifications occurring in diseases with or without treatments [3]. Proteomic capabilities are constantly evolving as a result of technological and methodological advances in the field, and as such, this provides powerful tools for the study of cancer research.

Proteome profiling studies have been granted the ability to reveal diverse molecular signatures and signaling pathways to monitor cancer progression and to assess the efficiency of therapeutic treatments [4,5]. A variety of quantitative proteomics methods have been developed, including label-free metabolic labeling and isobaric chemical labeling [6,7]. The integration of these techniques into oncological studies often uses multiplexed isobaric mass tags to label peptide digest mixtures and has proven to be an effective method in identifying potential targets of cancer aggressiveness and behavior growth. These quantitative proteomics studies can provide insight for developing novel therapeutics in future clinical settings. Taken together, the information provided by proteomics allows for understanding the biology, and it pinpoints the altered signaling complex that either interferes with or promotes tumor progression. Distinguishing anomalies in patients’ tumor profiles creates individual-specific cancer-related diseases, thus opening the avenue for personalized therapeutics.

Large-scale datasets can be generated from a single proteomics analysis, which gives rise to detailed information about the biological events surrounding tumor vitality and aggressiveness [7,8]. Hence, this information can be used to directly target a particular pathway of interest during treatment applications in translational studies. In this review, we will direct our focus to clinically prepared samples for translational research and recent potential biomarkers, as well as an in-depth analysis of the mechanistic signaling affecting cancer [4,8]. In addition, we will acknowledge current advancements in predominant malignancies via proteomics, with special emphasis on co-morbidity cases such as HIV-associated malignancies. 

## 2. Biomarker Discovery in Cancer Proteomics

**Figure 1:**
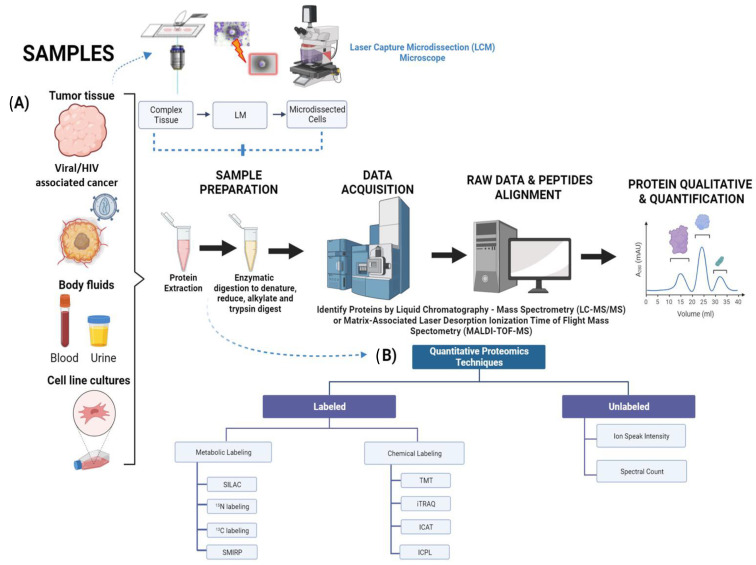
Diagram depicting quantitative proteomic workflow using tumor and HIV-infected tissue samples. (**A**) Flowchart illustrating proteomic strategies for biomarkers discovery in clinical cancer with three main types of samples: tumor tissue, body fluids, and cell line cultures. Tumor samples are micro-dissected using LCM microscope. Individual cells are harvested from a complex tissue in situ, lysed, followed by protein extraction/quantification, electrophoresis/staining to protein digestion, peptide recovery, and mass spectrometry. (**B**) Summary of prominent quantitative proteomics techniques that are used in sample preparation. Unlabeled methods consist of ion peak intensity and spectral counter, while labeled techniques are classified by metabolic and chemical labeling. Image created with BioRender.

Cancer biomarkers have transcended current treatments based on their validation and efficacy. In recent years, there have been promising advances in quantitative proteomics methodologies that play a major role in detecting novel cancer biomarkers. Quantitative proteomics operates under the principle that a signal response from any given analyte is related to its abundance within the mixture [9,10]. The implementation of a strong quality control and maneuvering of samples is pivotal to ensure data acquisition (Figure 1A,B). In this review, an overview of three main types of samples—tissue, blood and urine—originating from cancer patients and healthy individuals will be discussed. 

A key aspect of the proteomics process is the preservation of the tissue samples. There are three main methods to preserve these: fresh frozen (FF) [11], formalin-fixed paraffin-embedded (FFPE) [12], and optimal cutting temperature-embedded (OCT) [10,13]. Samples preserved through the FFPE method are known for providing clinical follow-up and are therefore considered a strong source in the field of proteomics, with emphasis in clinical settings. Meanwhile, the FF method is preferred since it can provide better proteome coverage. Previous studies have demonstrated that both FF and FFPE have strong precision when it comes to total protein concentration [12]. Colon adenoma research studies have focused on providing equivalence for proteomes coverage obtained from FF and FFPE tissue specimens using shotgun proteomics [14]. Other recent studies have confirmed that the improvements in terms of sample preparation have conducted to more efficient de-crosslinking, thereby allowing overall protein species to be identified for the digestion process to occur [15]. The determinant part of the experimentation that leads to a better outcome for biomarker validation is the handling of samples, due to its efficiency and accuracy in the purification process. Laser Capture Microdissection (LCM) represents a useful tool for the separation and purification of cells used in a given study. Its usage consists of analyzing the broad range of cancer-mediated signaling as well as metastatic tendencies that can arise [16,17]. First, cells of interest are required to be fixed to maintain and not disrupt cellular protrusions. Second, the cells are separated through lasers with the purpose of melting the thermo-film that surrounds the capping structure (Figure 1A) [18]. After successful cap removal, the tissue samples adhere to this cap.

To compare the sample effect between FF and FFPE, two studies will be discussed in which the researchers aimed to compare the performance of paired FF and FFPE tissues in both genomics and proteomics experiments, respectively. The objective of both studies was to determine which tissue type yielded better results in terms of genomic and proteomic analysis. The experiments involved obtaining paired FF and FFPE tissue samples from the same individuals to evaluate their performance. The first study to be discussed focused on examining protein retrieval in different types of kidney tissues from human biopsies [19]. Initially, the researchers analyzed 15 µm thick formalin-fixed (FF) kidney tissues and compared the results obtained using various protocols. They found that the highest number of identified proteins (630 ± 80) was obtained from 15 µm FF tissues using a buffer containing Rapigest. This was followed by ProteaseMax (498 ± 51) and PPS (297 ± 188) buffers. Subsequently, they applied these developed protocols to 4 µm thick FF and FFPE (formalin-fixed paraffin-embedded) kidney tissue sections. In the case of 4 µm FFPE tissues, the buffer with PPS yielded the highest protein recovery (265 ± 44). Since FF tissue samples results were higher than FFPE, this demonstrates that FF is better than FFPE in terms of protein quantification and identification. Furthermore, their findings indicate the similarity in physicochemical properties of proteins extracted from 4 µm FF and FFPE tissues, further supporting the appropriateness of the in-solution protocol used in this study. Overall, the study concluded that FF tissues are superior to FFPE tissues in proteomics experiments. The findings highlight the importance of using FF tissues when high-quality genomic and proteomic data are required.

In terms of the genomic study, the researchers aimed to evaluate the feasibility and reliability of microarray studies analyzing transcriptome data from FF and FFPE tissues obtained from striatal areas of post-mortem rat brains and hippocampus biopsies from humans [20]. The whole-genome analyses demonstrated a high degree of agreement between FF and FFPE arrays. However, when examining individual genes, a higher variability in data was observed in FFPE arrays compared to FF. The researchers attributed this variation to the inherent fragmentation of RNA materials in FFPE tissue samples caused by formalin fixation. Consequently, the use of FF tissue samples resulted in more-precise and accurate results. Furthermore, FFPE tissue blocks, despite exhibiting strong in-situ hybridization signals, do not necessarily yield high-quality RNA, leading to limited meaningful data in downstream experiments [20]. Overall, results demonstrated that FF tissues outperformed FFPE tissues in both genomics and proteomics analyses. In the genomics experiments, FF tissues showed higher-quality RNA microarrays with less degradation compared to FFPE tissues. This allowed for more accurate and reliable genomic profiling. Similarly, in the proteomics experiments, FF tissues exhibited better protein retrieval compared to FFPE tissues. The FF samples yielded a higher number of identified proteins with less variability, indicating the better preservation and extraction of proteins.

Among some of the commonly-used screening techniques are: Matrix-Associated Laser Desorption Ionization Time of Flight Mass Spectrometry (MALDI-TOF-MS) [21], multiplex quantitation using isobaric chemical tags such as Tandem Mass Tag (TMT) [22], Isobaric Tag for Relative and Absolute Quantification (iTRAQ) [23], Isotope-Coded Affinity-Tag-based protein profiling (ICAT) [24] and Isotope-Coded Protein Label (ICPL) [25] for cell culturing and the collection of samples in the form of biofluids (Figure 1 and Table 1). The labeling methods described previously have their own strengths and weaknesses ranging from cost effectiveness to limitations in project design (Table 1). In this review chapter we will focus on the usage of these proteomic tools. Isobarically labeled TMT peptides in protein digests, which can arise through treatment, allow us to quantify protein species, proteoforms, and proteome complexity [26]. Each TMT tag is composed of four regions. There is a protein reactive group that will bind to peptide samples and a mass normalizer group that serves to stabilize or normalize the isotopes of carbon and nitrogen, so the entire tag weights the same. This is followed by a mass reporter group, which when fragmented during the second-stage mass spectrometry (MS/MS) will yield a reporter of a different mass, and finally, there is a cleavable linker, which is cleaved by higher-energy collision dissociation (HCD) during MS2 and yields the reporter ion [26,27,28]. In relation to iTRAQ, this technique allows multiple samples to be processed at once (up to eight) in one experiment through the use of peptide labeling [29]. The effective integration of iTRAQ has also contributed to research in HIV-1 latent infections with cluster of differentiation 4 (CD4)-positive cells. This has allowed the detection of altered protein species and signaling complexity that arises in HIV-1-infected CD4+ cells, including oncogenic disturbances [30]. The incorporation of iCAT into quantitative proteomics has been demonstrated to be a precise tool to measure the proteoform changes for quantification purposes. This technique is known for its convenience of use in any given sample of organisms and its ease of use. However, its usage is limited to cysteine residues in peptides. Similarly, the ICPL labeling tool benefits protein species rich in lysine residues and also holds N termini. ICPL has demonstrated precise high-sequence information [31,32]. The most common methods used to detect biomarkers are labeled and unlabeled quantitative proteomics techniques. The labeled approach includes metabolic and chemical. A few popular examples of metabolic techniques are Stable Isotope Labeling by Amino Acids in Cell Culture (SILAC) [33], ^15^N and ^13^C labeling [34], and Subtle Modification of Isotope Ratio Proteomics (SMIRP) [35]. SILAC is considered a precise method due to its efficiency in labeling samples as well as overall preservation of protein species [36]. ^15^N and ^13^C labeling isotopes are supplemented to the growth media with the goal of integration into an organism, tissue, or cells. Lastly, SMIRP is known for its convenience of implementation in-vivo in full organisms, which include plants, bacteria, yeast, drosophila, and ultimately mammals such as mice and rats [31,35].

Liquid Chromatography coupled to tandem Mass Spectrometry (LC-MS/MS), multiple reaction monitoring, and Sequential Window Acquisition of all Theoretical Mass Spectra (SWATH-MS) methods are proteomic technique additions that require personnel with a broad knowledge of their operation (Figure 1) [37]. The signal intensity in LC-MS/MS emitted from electrospray ionization has been reported to highly correlate with ion concentration. This indicates that relative peptide levels in the samples can be determined directly from the intensity peaks. Since these experiments provide a large amount of data, sensitive computer algorithms such as MaxLFQ [38] or iBAQ [39] are required for automated ion peak alignment and comparison. 

**Table 1:** Comparison between each screening and labeling method used in quantitative proteomic.

Screening/Labeling Tools	Advantages	Drawbacks	Expenditures
MALDI-TOF-MS [40]	Easy to setup and analyzed	Poor sensitivity towards some bacteria specie such as Shigella and *E-coli*	Low cost
	Relatively high sensitivity for detection of closely related microbial species	Not suitable for detecting small numbers of bacteria in pathogenic and sterile research	
TMT [41,42]	Allows for identification of different samples with greater ease Increased sensitivity to phosphopeptides in reverse phase liquid chromatography Enable multiplexing of up to 18 samples simultaneously	Fragmentation of peptides may result in chimeric M/S spectra, which can lead to incorrect protein/peptide fold changes	High cost
iTRAQ [31,43]	Versatile tool that can be applied to a wide range of samples and organisms such as in vitro and in vivo.	High variations when implementing at the peptide level	Varies on sample numbers (High or low cost)
	Fast method	Quantification tends to be rather poor due to small amounts of mass spectra involved (usually one or a few more)	
	Enables multiplexing of up to 8 samples simultaneously	Requires a mass spectrometer that can analyze the low regions of m/z	
ICAT [31,44]	Can be applied to any sample type Fast method	Mild variations when implementing at the peptide level Only specific to Cys residues in peptides	High/Low cost
ICPL [25,31]	Can be applied to any sample type Fast method	Mild variations when implementing at the peptide level Only specific to Lys residues in peptides and protein N terminus	High/low cost
SILAC [31,33]	Can be implemented to an organism with relative ease and low variations High sensitivity and precision High preservation of proteins	Fails to be incorporated to samples relating to humans Slow method	High cost
^15^N [31,34]	Low variation when implementing in organism Does not discriminate between peptides. Therefore, it can be incorporated to any sample	Fails to be incorporated to samples relating to humans Slow method Unable to identify molecular weight before each peptide be subjected to identification	High cost
^13^C [31,34]	Low variation when implementing in organism Does not discriminate between peptides. Therefore, it can be incorporated to any sample	Fails to be incorporated to samples relating to humans Slow method Isotopes can inhibit identification and quantification	High cost
SMIRP [31,35]	Low variation when implementing in organism Can be incorporated to a wide variety of in-vivo organisms Does not discriminate between peptides. Therefore, it can be incorporated to any sample	Fails to be incorporated to samples relating to humans Slow method	High/low cost

Overall, tissue-based proteomics strategies have been successfully applied to many cancer types, including predominant forms such as breast, prostate, and lung [4]. The tissue sample analysis provides the most accurate information about the tumor’s physiological state and the LCM technology has continuously enabled significant proteome coverage with validated quantification. In addition, using isolated organelles for protein extraction has been demonstrated to significantly reduce the complexity of the extracted proteome. Generically, proteomics is defined by two main approaches: top-down [45] and bottom-up [46]. Top-down proteomics is the resolution of intact protein species and can identify and quantify unique proteoforms through the analysis of intact proteins, while bottom-up proteomics consists of the peptide mass spectrometry (MS) of proteolytic digests. 

## 3. Prospects of Diagnostics and Therapeutics of Proteomic Research in Predominant Cancers including HIV-Associated Malignancies

Proteomics-based analysis plays a fundamental role in tumor diagnostics and therapeutics by discovering potential biomarkers to detect early stages of cancer and inhibit carcinogenesis [47,48]. The scarcity of precision and personalized medicine in anti-cancer therapy contributes to conjure resistance and survival [49]. Some intrusive cancer types make them difficult to diagnose and treat due to their innate oncological settings. In addition, patients suffering with cancer-enhancing diseases prove more difficult to treat based on current limited knowledge. Here, we discuss the recent advancements of proteomics in six clinically challenging cancer pathologies: breast, lung, retino, prostate, glioma. and HIV-associated malignancies. Moreover, we will be discussing current limitations and challenges of proteomics in clinical application and possible future clinical integrations.

**Triple-negative breast cancer (TNBC)**: This is considered a more aggressive variant in comparison to other breast cancers as it lacks the expression of the usual estrogen receptors (ER), progesterone receptors (PR), or Her2/neu receptors that are usually targeted by chemotherapy treatment [50]. While genomic classifications are used to identify subtypes of breast cancer, they are not always very useful in guiding treatment choices due to significant heterogeneity in aggressive subgroups of TNBC [51,52,53,54,55,56,57]. To better understand the molecular heterogeneity of TNBC, Reverse Phase Protein Arrays (RPPAs) were attempted as the first large-scale proteomic characterization to measure cancer-related protein expression and phosphorylation activity [51,52]. The results provided key insights into the heterogeneity at the molecular level with limited availability in data. In addition to RPPA, Peptide-level Expression-Change Averaging (PECA) was also used to identify protein biomarkers associated with clinical outcomes in TNBC. By using Cox proportional-hazards analysis on protein abundance and survival outcomes, researchers were able to identify 85 proteins pertaining to a longer Recurrence Free Survival (RFS), including immune-related proteins, and 18 proteins with poor RFS [51]. This study utilized a highly sensitive mass spectrometry-based methodology called Single-Pot, Solid-Phase-enhanced, Sample Preparation-Clinical Tissue proteomics (SP3-CTp), which was able to capture important biological features in FFPE tumor samples [51,58,59,60,61].

Immunotherapy has been attempted in patients with breast cancer but has had limited benefit to individuals expressing mutations in Programmed Death Ligand 1 (PD-L1) and Tumor Mutational Burden (TMB) due to poor sensitivity and the specificity of current methods [62]. Recently, a group of researchers (Cun and colleagues) proposed, developed, and tested a Tumor Immune Risk Score (TIRS) using eight potential biomarkers of TNBC that resulted in a correlation with survival prediction. In addition, they identified treatment guidance through four different Tumor Immune Microenvironment Types (TIMTs) that required further validation [62]. Lastly, relating to breast cancer, Adenoid Cystic Carcinoma (ACC), a rare indolent malignancy, has overlapping features with TNBC that can lead to unnecessary treatment via surgery or chemotherapy [63,64]. To provide a method to differ both neoplasms, a recent study compared protein expression in ACC and TNBC via mass spectrometry-based proteomics. This has resulted in substantially different proteomic profiles seen in immune response, vesicle-mediated transport, unregulated proteins, and ribosome biogenesis. These results may offer a deeper understanding of both biological behaviors of both neoplasm and potential target therapy [64]. 

**Lung adenocarcinoma (LUAD**): The need to identify a biomarker for LUAD to provide early detection and treatment that responds appropriately to the neoplasia is essential. A review of the literature of the subject has suggested the use of cytokines as biomarkers for lung cancer due to the correlation between expression and lung cancer progression [65,66]. Their role in disease progression and survival make them attractive candidates for oncogenic studies. Markers of therapeutical use include Vascular Endothelial Growth Factor (VEGF) coupled with angiogenesis; Tumor Necrosis Factor (TNF)-alpha associated with tumor suppression; Epidermal Growth Factor Receptor (EGFR); and Transforming Growth Factor (TGF)-beta expressions linked with either tumor survival or apoptosis. Specific targeted approaches along with other pharmacological limitations could be realistically implemented in LUAD clinical research practice with the advancements of proteomics [47]. Xu et al. carried out a comprehensive proteomics analysis of 103 cases of LUAD, the most common of non-small-cell lung cancers and most common subtype in non-smokers [67]. Therapeutic management to this day is mainly surgery and chemotherapy, although recent advancements in targeted therapies against oncogenic driver mutations have achieved remarkable success [68,69,70,71,72]. Inhibitors against EGFR, Anaplastic Lymphoma Kinase (ALK), and B-Raf proto-oncogene (BRAF) V600E mutation, as well as immune checkpoint inhibitor antibodies against PD-L1, have been approved for the precision treatment of LUAD [73,74]. Despite this progress, lung cancer is the second-most-common malignancy and remains the prime cause of cancer-related death worldwide, with LUAD accounting for 63% of this [67,75]. This is largely due to lack of known genetic alterations in the key oncogenic signaling pathways and the difficulty of targeting oncogenic mutations (KRAS mutations) [67,76,77]. Additionally, a lack of early detection and diagnosis markers contributes towards this disease [47]. The proteome-based stratification of LUAD will lay a foundation for understating the heterogeneous nature of the disease and for developing new therapeutic approaches. An MS-spectrometry-based label-free quantification strategy has been adopted and several proteins, including Heat-Shock Protein 90B (HSP90B) (HSP90AB1), were identified as prognostic biomarkers for LUAD [67]. The proteomic results from these studies highlighted that HSP90ß was overexpressed in tumors. The upregulation of HSP90ß is associated with poor prognosis and was also observed in patients with a co-mutation of EGFR and TP53. To confirm the potential value of HSP90ß as a prognostic marker of LUAD, Enzyme-Linked Immunosorbent Assay (ELISA) was applied to examine the plasma protein level in an independent cohort of 705 LUAD patients and 282 healthy controls. In addition, HSP90ß was downregulated in S-1, with the best prognosis. This proteomic result correlated with the three subtypes (S-1, S-II, S-III) related to histological, clinical, and molecular features [67].

**Prostate cancer (PCa)**: **PCa** is the most common cancer among males and the second leading cause of death [78]. This is due to the strong correlation between malignant and non-malignant prostatic epithelial cell secretion, which can lead to an elevated number of false positives detected and overdiagnosis during screening. To diagnose PCa, the measurement of Prostate-Specific Antigen (PSA) levels, Digital Rectal Examination (DRE), and radiological imaging, along with a histopathological analysis of tissue biopsies are methods commonly used [79]. While PSA screening is available for prevention, its specificity is limited, and the medical community remains divided on its use. To overcome this, researchers have suggested the incorporation of additional clinical biomarkers [80]. A recent study found that a combination of plasma protein species, including PSA, hexokinase 2 (hK2), microseminoprotein-beta (MSMB), and Macrophage Inhibitory Cytokine 1 (MIC1), performed better than PSA alone [75]. The use of proteomics sequencing and methodologies such as ELISA, protein microarrays, aptamer-based assays, and MS-based proteomics have provided insights into effective biomarkers for PCa [75,76,77,78,79,80,81,82]. Other approaches such as Data Independent Mass Spectrometry and Multiple Reaction Monitoring Spectrometry (DIA-MS+MRM-MS) have identified potential PCa biomarkers, such as the recent 75-protein panel that can differentiate between PCa and benign prostate hyperplasia (BPH) [80,81,82,83,84,85,86,87,88,89]. However, the proteome may not always translate aberrations in PCa, which gives way to the understanding of molecular signatures [90,91,92]. The heterogeneity of PCa signifies that gene expression can vary in different regions of the same carcinoma. Overall, proteomics sequencing has been an integral part in PCa research and has revealed insights into the transcriptional regulation of RNA and studies of protein complexity [90].

**Glioblastoma Multiforme (GBM)**: GBM is the most lethal form of brain cancer, one of the most malignant of all tumors [93]. Its diagnosis and treatment are often complicated by the tumor’s complex microenvironment characterized by heterogeneity and immunosuppression. Consequently, determining its various subtypes can be challenging. However, proteomics-based research is crucial for the future diagnosis and treatment of GBM [94]. Despite multiple obstacles, proteomics can potentially accelerate GBM research regarding its diagnosis and treatment via immunotherapy. One study developed a proteomic atlas using LC-MS/MS, which aligns abundance levels of 4794 protein species for the identity of histomorphological features and niche-specific biomarkers. Previous efforts focus on specific molecular signatures compared to tumor growth markers [95].

Due to the absence of strong pharmacological treatments, the identification of early biomarkers is important to improve survival rates among individuals. Recent tools in molecular biology can be attributed to multi-omic techniques, among the most important being the quantitative proteomic for analysis of protein complexity [96]. GBM still remains a persistent threat with low survivability. Treatment alternatives, including chemotherapy agent temozolomide, increase this rate further by a few months and remain effective for the treatment of high-grade glioma [97]. Modern studies have focused on sophisticated chips with high sensitivity, with the goal of using them as the first bio-printed cancer on-a-chip. The aim is to attempt to reproduce the patient’s therapeutic responses using these chips [98]. However, despite these advances, these therapies have limitations since they fail to completely reproduce the working brain of a patient, according to the Barrow Neurologic Institute [99]. 

**Retinoblastoma (Rb)**: **Rb** is the most common form of pediatric ocular cancer type [100], and while current treatment options have improved, metastasis to the central nervous system remains an obstacle for prevention [101]. Immunotherapy against Rb may offer a treatment option that preserves vision and enhances patients’ life modalities; however, current insights into the tumor microenvironment remain limited [102]. Recent studies relating to proteomics and Immune Cell Infiltration (ICI) have demonstrated two types of gene subgroups responsible for tumor metastasis. Furthermore, hypermethylation has been identified as a potential mechanism for treating Rb, as it regulates the TIMT in the high-ICI groups [103]. Biopsies have long been considered unsafe practices with a high-risk of ocular spread in Rb, insisting that safer approaches are needed for therapeutic management [104]. Endophytic tumors that can be present in the vitreous, or have a mixed presentation, remain a strong obstacle when handling intraocular sections of Rb [104,105], where the enucleation of the affected eye may be the only option for advanced tumors [104,106].

Research has demonstrated that exosomes act as promising biomarkers compared to other biopsy targets for diagnosis and tumor detection in Rb. The use of a single-shot LC-MS/MS analysis and comparisons analysis revealed a total of 99 and 35 proteins exclusively present in Rb tumor exosomes. These protein species that serve as a potential source of diagnosis include: Abl Interactor 2 (ABI2), Glutathione S-Transferase Mu (GSTM), Neurocan (NCAN), and V-type proton ATPase subunit C 1 (ATP6V1C1) [104].

**Figure 2:**
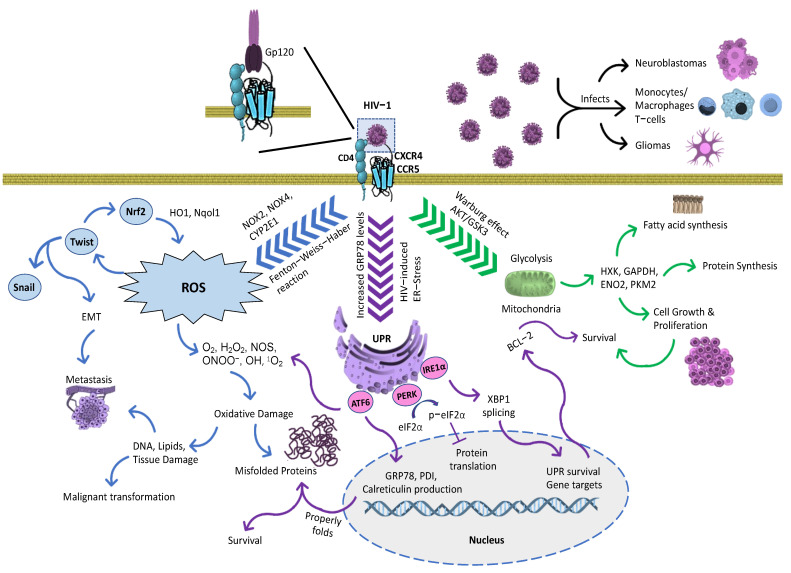
Mechanistic representation of viral malignancy effects triggered by HIV-1 gp120. HIV-1-infected cells infect by using gp120 capacity to induce oxidative, ER stress and glycolytic responses. Gp120 increases free radical production from monocytes and macrophages, which can induce ROS. In astrocytes, ROS production is enhanced using several signaling methods, specifically with cytochrome CYP2E1, NOX2 and NOX4, and the Fenton–Weiss–Haber reaction. ROS-mediated gp120 effects directly activate Twist [145], subsequently modulating Nrf2, and thereby stimulating antioxidant enzymes such as HO1 and Nqol1. In addition, Twist leads to the regulation of Snail, which serves as a transcriptions factor. Both transcription factors, Twist and Snail, are involved in epithelial-to-mesenchymal transduction (EMT), inducing metastasis. The effects of ROS extend towards DNA, lipid, and tissue damage in neighboring cells. Therefore, DNA damage promotes the malignant transformation of normal cells while propagating cancer cells [146]. Gp120 also increases the rate in glycolytic metabolism leading to proliferation, survival, lipid, and protein synthesis [117]. In addition, gp120 triggers ER stress to correct the proper folding of proteins, subsequently activating UPR markers [118]. Figure arrow legends: blue arrows—Process leading to ROS; purple arrows—UPR and ER stress-induced pathways; and green arrows—Metabolic response triggered during HIV-1 infection.

**Figure 3:**
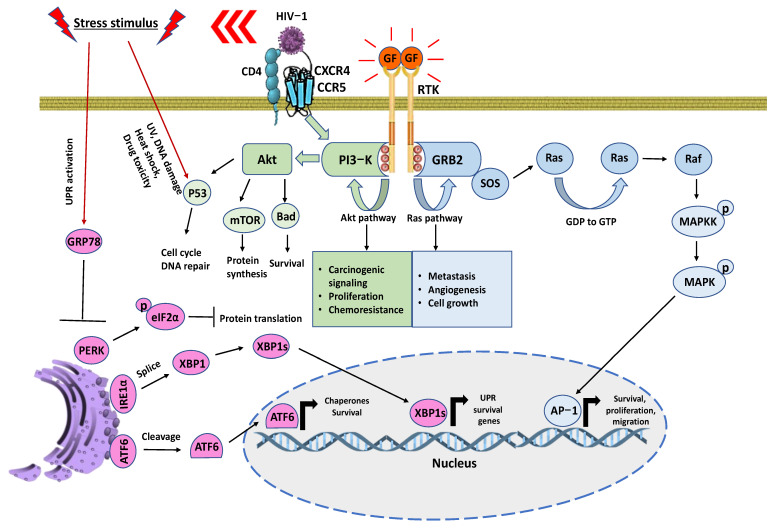
Schematic representation of signaling events for oncological and HIV-associated malignancies. RTKs are crucial regulators of cancer survivability while also corresponding to drug resistances. This allows recruiting adaptor proteins to signal into branching pathways. Mainly the Akt and Ras. CD4+ cells can influence cell survival through CXCR4/CCR5. There is an activation of multiple phosphorylated tyrosine residues that serve as docking platforms through the phosphotyrosine binding (PTB) domain of RTK to regulate a wide range of signaling complexes that benefit cancer-mediated growth. Simultaneously, stress factors such as HIV-1 infection induces UPR and leads to the recruitment of GRP78 for proper folding [117,118]. The endoplasmic reticulum sensors PERK, IRE1α, and ATF6 are no longer inhibited by GRP78, which halts protein translation, the splicing of X-Box Binding Protein 1 (XBP1), and the cleavage of ATF6. The precedence for these oncogenic and metabolic activities influences the tumor microenvironment and results in a favorable cycle of proliferation, angiogenesis, chemoresistance, metastasis, and immune evasion.

**HIV/AIDS-associated Malignancies**: HIV-1 infections have been linked to tumor advancements. Statistically, patients have been reported to be 20–70 times more likely to develop non-Hodgkin lymphomas as well as 10 times more likely to develop Hodgkin lymphoma. With the rise of cancer cases in HIV-infected patients, there has been an urgent need for the development of HIV-specific biomarkers. The indirect effects of these viral infections can extend towards more predominant cancer disparities such as the lungs, where patients have been known to be at least three times more likely to suffer [107]. Proteomics studies aim to address the search for successful biomarkers and to decipher the mechanistic interactions between co-morbidity cases that can aid in future therapeutics for HIV-infected patients. The HIV-1 envelope and internal replicative proteins are the main culprits for cancer-inducing properties by interacting with the known signaling molecules highlighted in Figure 2 and Figure 3. Specifically, the main effects of HIV-1 in cancer can either be a direct or indirect cause for the transformation of these cells [108]. Throughout this review, current knowledge regarding HIV-1 infection and the signaling outcomes being affected in cancer cases are discussed. The first proteomic characterization of co-morbidity studies with GBM HIV-infected patients was performed using patient tumors in comparison to normal tissues. To obtain a comprehensive proteogenomic characterization, samples of a Whole-Exome Sequencing (WES) were performed. Pairing transcriptomic and proteomic data from the patients created 6203 mRNA–protein pairs [95]. Consequently, this has linked genes involved in metabolic processes with positive mRNA–protein correlations [109,110]. In addition, PYCR2 and ADH1A, involved in amino acid metabolism and oxidoreductase activity, respectively, were affirmed as representative prognostic proteins using tandem mass spectrometry MS/MS, thus revealing potential biomarkers for clinical application [109,111,112]. Moreover, a recent investigation using protein–protein network analysis has discovered approximately 40 differentially regulated proteins of interest to identify useful markers for HIV-1 co-morbidities [113]. Other proteomic studies extended their research by identifying novel signaling for personalized medicine in HIV-infected patients with a range of cancer types. Among these molecular signaling targets, researchers have focused on migratory and cytoskeletal components such as cofilin1, cystatins B, and L-plastins [30,114,115]. Other proteomic studies focused on identifying the relation between transcriptional behaviors on cellular structures as potential biomarkers in HIV-1 malignancies [116]. Additionally, there has been a growing interest highlighting the scientific merit of clinical outcomes related to co-morbidity between GBM and HIV-1 research. Specifically, for the first time, scientists demonstrated that HIV-1 envelope glycoprotein 120 (gp120) significantly increased fatty acids, protein synthesis, glycolysis and ER stress/Unfolded Protein Response pathways in glioma, contributing to cancer survival and proliferation [117,118]. Subsequent future discoveries of novel proteomic signature targets are expected to lead to more effective and specific therapies to reduce the disparities of malignancies in the HIV-infected population. With a successful transition of proteomics to the clinical setting and studies, the advancement of precision oncology and personalized medicine will be that much more.

## 4. HIV/AIDS Malignancies in CD4+/CXCR4/CCR5-Infected Cells including GBM

People living with HIV-1 are at increased risk of developing cancer despite successful antiretroviral therapy [107,119,120,121]. Multiple malignancies can be attributed to HIV-1, however, some of the most common neurological malignancies are primary central nervous system (CNS) lymphoma and GBM. While the development of GBM is an uncommon complication in HIV-1 patients, the introduction and implementation of HAART has resulted in a decrease in AIDS-defining malignancies and a subsequent increase in non-AIDS-defining malignancies such as the mentioned GBM [122,123,124]. A review of cases and studies has shown that age and CD4 counts are not correlated to survival and tumor progression as an indicator of survival in patients with HIV-1 and GBM [122]. There are two proposed mechanisms as to how HIV-1 may cause GBM: one is via immunosuppression, which has been directly linked to GBM by a mechanism of decreasing anti-tumor activity and thus promoting the development of malignancies; and the other mechanism is via HIV regulator genes such as negative factor (Nef) and trans activator of transcription (Tat). Other mediators can be attributed to cytokines such as interleukin (IL)-1, IL-6, IL-8, and TNF-alpha, which have been recognized in studies to have transforming properties in neural stem cells or astrocytes. This can initiate their differentiation into glioma or malignant cells from AIDS-associated malignancies and thus could be contributing to the development of GBM [122] However, a cross-sectional study by Luxwell et al., of the prevalence of HIV in glioma patients in sub-Saharan Africa, indicates that the prevalence of HIV in glioma patients is decreasing when compared to the general population as described previously in a study in Mexico and Brazil [124]. The authors describe and associate the observations to a phenomenon labeled the “antiglioma effect”, where HIV, HAART, or both can be responsible for being protective from gliomas; this would require more studies for insight [124]. Here, we will suggest novel mechanisms behind this phenomenon. Moreover, we cover current knowledge regarding HIV-1 malignancies through viral envelope protein and host cell interactions. HIV-1 has been extensively studied to infect CD4+ cells using CXCR4 and CCR5 as co-receptors to gain entry [125]. CXCR4 and CCR5 are chemokine receptors and members of the G-protein-coupled class family; they have the role of mediating leukocyte development, trafficking, angiogenesis, and immune response [126]. CXCR4 and CCR4 are attractive candidates for cancer studies since they have been reported to modulate physiological processes associated with proliferation, survival, tumor growth, invasions, and epidermal growth factor receptors [127,128]. CXCR4 has been a consistent marker throughout predominant oncological diseases, including lung cancer [129,130] and PCa [131]. Interestingly, the upregulation of CXCR4 has been linked to an increased rate of migration in lung cancer towards the bone marrow [129]. The significant role of CXCR4 in cancer development has led to this receptor being implemented as an additional prognostic marker for both lung cancer and PCa patients [132,133]. Regarding its oncological effects in some of the predominant cancer types, it has previously been postulated that HIV-1 interaction with CXCR4 can render CD4+ cells vulnerable to malignant transformation [134,135]. As a result, the overexpression of key signaling pathways (Figure 2 and Figure 3) can open the possibility for proliferative triggers to activate prematurely or when not needed. Similar to the effects of CXCR4, CCR5 is ubiquitously expressed in tumors. Its overexpression has been seen in PCa [136] and Hodgkin’s lymphoma [137], among other cancer types. Through this, CCR5 has been postulated to be a prognostic marker throughout lung cancer and PCa due to its overexpression. However, common CCR4-specifc ligands such as CCL5 show poor prognosis in these cancers [137]. Having said this, both HIV-specific co-receptors CCR4 and CCR5 have ties to tumor tendencies and share similar oncological pathways. 

Protein species relating to HIV-1 can interact with oncological signatures from other cancerogenic viruses. This is responsible for the bulk of oncogenic disturbances to individuals suffering from HIV/AIDS. Specifically, HIV-1, gp120, Tat, Nef, matrix p17 and Reverse Transcriptase (RT) have been directly linked to malignant tendencies. ER stress and proliferative routes research conducted by the groups led by Boukli and Kucheryavych demonstrated that the entryway protein gp120 activates downstream UPR/ER stress signaling [118] as well as glycolysis [117]. The cancerogenic properties of these overlapping signal complexities are reported to affect neighboring epithelial cells, which can result in malignant transformation (Figure 2). 

Some of these characteristics can be attributed to the survival tendencies that UPR conjures by upregulating pro-survival proteins [138]. This can lead to the activation of B-cell lymphoma 2 (BCL-2) family proteins in the mitochondria that have a strong impact on cell survival (Figure 2) [139]. This process is triggered by the inhibition of apoptosis, autophagy, and cell cycle modulation. Consequently, this signaling overlaps with the glycolytic pathway and promotes a stronger response by regulating energy metabolism and proliferation through the Warburg effect [140,141]. Glycogen Synthase Kinase-3 (GSK3) contributes to anti-apoptotic roles by modulating metabolism and is controlled through the Akt/PI3K pathway in an inversely proportional manner [142]. Co-morbidity cases between HIV-1 and GBM have demonstrated that GBM growth and proliferation are affected through this signaling. It has also been shown that key metabolic markers such as Hexokinase (HXK), Glyceraldehyde 3-phosphate Dehydrogenase (GAPDH), Enolase 2 (ENO2), and Pyruvate Kinase M2 (PKM2) play an important role as downstream effectors of mitochondria activity [117]. Therefore, the metabolic functions of cancer cells are manipulated through Akt/PI3K/GSK3 signaling (Figure 2). HIV-associated malignancies can also be attributed to an increase in reactive oxygen species (ROS). The Fenton–Weiss–Haber reaction is partially associated with this response by converting hydrogen peroxide (H_2_O_2_) into a hydroxyl radical (^•^OH) [143] via the NOX pathway. ROS-mediated damage further activates transcription factors such as Twist and Snail, which simultaneously promote metastasis. While ROS induce lipid, DNA, and tissue damage during viral infection, the breakdown of DNA contributes to malignant transformation [108]. The accumulation of oxygen derivatives leads, on the other hand, to the accumulation of unfolded proteins, which in turn induce oxidative damage mitigated by proper protein folding, favoring survival. Lastly, it has been shown that the carcinogenic effects of HIV-1 gp120 can also extend to neuroblastoma cells, which have been shown to induce proline oxidase followed by pyroline-5-carboxylate and ROS generation [144]. Therefore, a change in cancer by the upregulation or downregulation of the above-mentioned proteins can result in tumor transformation and metastasis (Figure 2). All in vitro and in vivo studies are comparable to laboratory and clinical settings in AIDS-related malignancies, HIV-1 disease, and viral-induced tumors. These investigations aim to develop novel therapies to reduce the cancer disparities of GBM among the HIV-infected population. In this review, we dissected the roles of HIV-1 co-receptors on predominant cancer types during viral infection. In addition, we highlighted the potential of HIV-1 to trigger the malignant transformation of predominant cancer types, with an emphasis on GBM (Figure 2).

## 5. Signaling Pathways in Cancer: Mutual Crosstalk between ER Stress, Survival, Proliferation, Cell Cycle, and Migration 

Advancements in proteomic technologies have allowed for the identification of potential deregulated signaling pathways in tumor cells. With equal standing, they have been utilized to understand alterations in the host’s proteome to characterize the specific stages of diseases. Amongst the most common hallmarks that are beneficially deregulated in cancer are: cell cycle progression, proliferation, survival, angiogenesis, immunoregulation, and migration (Figure 3) [147]. With equal standing, the Unfolded Protein Response (UPR) and Endoplasmic Reticulum (ER) stress signaling also serve as common factors in a variety of cancer types [148,149]. These mechanistic signature pathways act as the premises for cancer progression. This review focuses on known signaling pathways that govern their deregulation, metastasis, and tumorigenesis, as well their signal promoters. We will also highlight cancer-inducing signaling triggered during HIV-1 infection and co-morbidity cases. Distinguishing cancer types has been linked to unique alterations in protein expression levels that allow cells to survive and over-proliferate in their oncological environment. The proportions of these properties overlap with other signaling complexes that come together and allow favorable tumorigenic conditions to take place. In the case of GBM, it uses the Wingless-type (WNT) pathway to aid in tumor formation and progression [150,151]. 

Evidence from a growing number of studies suggests that TNBC stem cells promote metastasis by regulating the WNT, Nuclear Factor kappa B (NFκB), Notch, Signal Transducer and Activator of Transcription 3 (STAT3) and hedgehog (Hh) pathways to induce an epithelial-to-mesenchymal transitional change [152,153,154,155,156]. Similar to GBM and TNBC, PCa shares signaling homology. In current clinical trials approaches, common cancer-promoting regulators such as the WNT, cyclin-dependent kinases and Ak strain transforming (Akt)/Phosphatidylinositol-3 Kinase (PI3K) pathways are being tested for PCa and other cancer subtypes, including the ones described above for treatment implementations [157,158,159]. In this review, we will also discuss lung cancer as the most predominant disease [160]. Its most extensively studied mechanism is the Receptor Tyrosine Kinase (RTK) and downstream effectors such as the small GTPase Rat sarcoma (Ras) and Mitogen Activated Protein Kinase (MAPK) [161]. Like other oncogenic alterations, lung cancer has genetic mutations, which in this case allow it to carry point mutation, resulting in constant RTK activation even in the absence of a ligand [162]. These oncological disturbances have the tendency to share two genetically altered mechanisms that includes Akt/PI3K/ Mammalian Target of Rapamycin (mTOR) and RTK/RAS/MAPK (Figure 3), among other complex structures. These receptors are activated by growth factor ligands with the purpose of inducing receptor dimerization, leading to a conformational change that auto-phosphorylates the intracellular Receptor Tyrosine Kinase domain. Subsequently, this will recruit adaptor protein Growth Factor Receptor-Bound protein 2 (GRB2) to allow for the Son of Sevenless (SOS) protein to tethered. As a guanine nucleotide exchange factor, SOS will increase the exchange rate of GDP to GTP, allowing the full activation of Ras. This activates downstream effectors such as Raf and the MAPKs, with the goal of inducing the transcription of proteins that favor migration, survival, and proliferation. In a similar manner, PI3-K is activated through RTK and allows it to phosphorylate the lipids into a third messenger that gives way to the activation of Akt. Once this outcome is fulfilled, the cell gains access to an extensive array of functions pertaining to protein synthesis, survival, and the modulation of the cell cycle [161,162,163,164,165,166]. As a result, this prompts the initial stages of downstream effectors responsible for controlling cell cycle progression, protein synthesis, and migration. In abnormal conditions, cancer takes advantage of these overexpressed signals in the form of uncontrollable proliferation, survival, and metastasis [163,164,165,166], respectively (Figure 3). 

Healthy and malignant cells that are subjected to stress-inducing factors can result in the disruption of proteoforms and folding complexity. This can be attributed to endoplasmic reticulum (ER) stress [138]. As a means to restore protein homeostasis, cells employ the Unfolded Protein Response (UPR) to properly correct misfolding that can arise in protein species. The survival tendencies of the UPR can be accredited to Glucose Regulating Protein 78 (GRP78), which is considered the master regulator of the UPR. Specifically, GRP78 binds to downstream effectors such as Activating Transcription Factor 6α (ATF6α), Inositol Requiring Enzyme 1α (IRE1α), and Protein Kinase R-like ER Kinase (PERK) and keeps them in an inhibited state under normal conditions [138]. GRP78 is a chaperone that senses and binds to unfolded proteins and configures them into a proper shape. During this process, all three downstream regulators (ATF6α, IRE1α and PERK) are activated. ATF6α and IRE1α are mostly involved in dictating cell fate with UPR-related survival genes, while PERK halts protein translation while giving priority to the UPR transcription [167] (Figure 3). The innate ability of the UPR to switch from a mild to a more severe form of cellular stress decides the efficacy and survivability of cells in terms of their ability to cope with ER stress response [168]. The survival tendencies of the UPR can indirectly overlap with oncogenic signaling (Figure 3); this can prove to be beneficial for the overall proliferation of cancer cells. However, the UPR can also trigger apoptosis if it fails to rectify the folding process of proteins for a prolonged period [169]. In a similar process, HIV-associated malignancies have been linked to the promotion of tumor growth. Research has shown that protease inhibitors lessen the progression of HIV-induced tumor development [170]. As a result, this blocks Akt-mediated signaling and renders cancer cells more susceptible towards radiotherapy [171]. The oncogenic properties of HIV-associated malignancies can be attributed to both of its entryway co-receptors (CXCR4 and CCR5) and cells that possess the CD4 receptor. The downstream effectors of these receptors can directly activate pro-survival and proliferative routes pertaining to the Akt/PI3K pathway, as seen in Figure 3 [172]. Lastly, HIV-1 infection is considered as a stress-inducer that can trigger UPR/ER stress mechanisms. Through these actions, it has been postulated that this process can serve to promote survival tendencies through the transcription of UPR genes [173]. 

**Figure 4:**
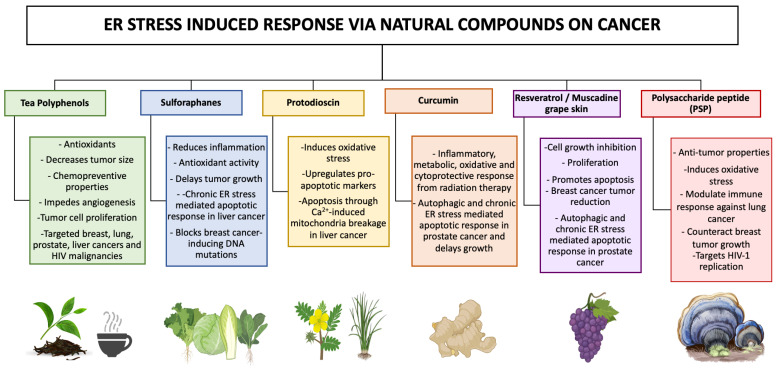
Natural compounds as treatments for cancer and HIV-associated malignancies using an ER stress-induced response. Cancer cells are being targeted using promising natural compounds as a mean of therapeutic treatment to revert cancer-mediated growth through chronic ER stress. Acute ER stress, on the other hand, promotes survival and proliferation in malignant cells. These compounds have unique anticancer potentials and are known for their beneficial properties, such as free radical scavenging, decreasing oxidative stress, and modulating inflammation.

Interestingly, natural compounds targeting ER stress response have been designed to kill cancer cells, with molecules showing excellent curative effects (See Figure 4). Here, we review recent advances in our understanding of the ER stress and UPR markers used to date [174,175,176]. Polyphenol teas have been shown to possess cytotoxic abilities on MCF-7 (breast), A549 (lung), PC3 (PCa), and HepG2 (Liver) cancer cell lines through the induction of cell cycle arrest [177]. Polyphenols have also been used as potent therapeutic agents in HIV-associated malignancies. Their anti-cancer effects are associated with a decrease in tumor growth, the inhibition of metastasis, and protection against carcinogens [178]. The natural extract from the turmeric rhizome plant, curcumin, has been demonstrated to induce chronic ER stress in PCa, subsequently promoting cancer death [179]. Similarly, muscadine skin extracts from red grapes have been used as a cancer treatment due to their ability to trigger UPR-mediated apoptosis in PCa [180]. Steroidal saponins such as protodioscin have also displayed anti-tumor effects on cancer cells through the promotion of ER stress-mediated reactive oxygen species (ROS). This compound has been reported to directly stimulate pro-apoptotic markers such as CHOP and mitochondrial breakage through ER-induced calcium influx [181,182]. Studies have shown the potential of sulforaphanes to be implemented as a natural therapeutic approach for breast cancer treatments due to its innate ability to induce cell cycle arrest [183]. In addition, sulforaphanes have been linked to triggering an ER-stress response, which is an anti-liver cancer property [184]. The manipulative strategies of HIV-1 infection with the objective of viral entry into the host cell have been known to specifically alter oncogenic pathways (Figure 2 and Figure 3). This outcome opens the opportunity for disease progression and cancerogenic properties. To combat these oncogenic and HIV-1-induced effects, researchers have been implementing proteomic approaches in combination with natural compounds with the goal to reduce cancer disparities. Namely, the natural compound polysaccharide peptide (PSP), derived from the mushroom of *Coriolus versicolor,* has been known to possess anti-tumor properties as well as to modulate the immune response against patients suffering from breast and lung cancer [185]. Particularly, it has the innate ability to activate an oxidative stress response and consequently counteract tumor growth [186]. Interestingly, PSP demonstrates the ability to extend its anti-cancer effects towards the immune system by inhibiting HIV-1 replication in THP1 and peripheral blood mononuclear cells through the upregulation of antiviral chemokines [187]. It has also been observed to target the entryway through overlapping signaling between UPR marker IRE1α and PKR in monocytes [188]. This review provides deeper insight into common overlapping pathways that are shared among predominant oncogenic disturbances as well as triggered during HIV-1 infections (Figure 3). Further, in this integrative review, we examine the literature on key compounds that are currently being researched as potential treatment options for combating cancer and HIV-associated malignancies (Figure 4). 

Ultimately, we will discuss the cell cycle and its unchecked growth. Tumorigenic cells frequently suffer genomic instabilities in abnormal proliferative environments [189]. This implication is further strengthened when external stimuli are involved; for example, stress overlaps with the hyperactivation of cell cycle regulator P53. The present phenomenon becomes apparent when mutations in p53 occur in over 50% of cancers. Under normal conditions, this protein is tasked with halting the progression of cell growth by acting as a transcription factor for control division [190,191]. It also serves a multitude of purposes, such as apoptosis, growth arrest, and sensing DNA damage under stressful conditions [191]. In a recent and detailed study, the use of quantitative proteomics was employed to identify the protein–protein interactions, and provided an insightful overview of possible mechanistic mutations that p53 suffers [192]. With all this stated, proteomics continues to serve as the leading premise in mechanistic studies involving cancer, and it also provides the opportunity for new therapeutic treatments to be implemented. Acquiring these new profound changes grants it the novel oncogenic properties term p53 gain-of-function [191,192]. 

## 6. The Role of Proteomics in Personalized Medicine 

Advancements during recent decades have increasingly recognized that no two cancer types are shared within individuals. While two oncogenic disturbances may be classified clinically and pathologically as the same, the response and resistance to treatment can be completely different. Although chemotherapy and radiotherapy have been first in line in the treatment of various cancers, the complexity and heterogeneity of cancer have led to the need for a personalized and precise approach for treatment [193]. By combining multiple disciplines, the advancements of new proteomics sub-disciplines such as proteogenomics are expected to provide screening tools. These findings, and particularly the interpretation of their results regarding specific genes and proteins of cancer patients, provide treatments options that target the proteins responsible for pathogenesis [37,193]. While precision oncology has been used for cancer prevention and screening, its main application has been the development of antineoplastic agents. As a result of these developed therapies, projects such as The Human Genome Project and The Cancer Genome Atlas paved new roads towards understanding the origin and causes of cancer by analyzing the genes that caused them [194]. Nevertheless, despite the remarkable success of genomic analysis, protein correlation between protein and mRNA expression in cancer showed that an integrated analysis of the proteome has the potential to uncover novel disease characteristics [193,194].

When compared to genomics, proteomics has been advancing rapidly and with promising results, including the development of drugs that directly target pathologies at the protein level instead of the gene level, such as inhibitors of MAPK, PI3K, serine/threonine protein kinase, BRAF, VEGF, and EGFR [193]. Drugs with said mechanisms of action have been essential in medicine for conditions such as V600E positive malignant melanoma, renal cell carcinoma, colorectal carcinoma, wet macular degeneration, and non-small-cell lung carcinoma. The development of the Clinical Proteomic Tumor Analysis Consortium has developed a comprehensive catalog of somatic mutations in cancer and led to reclassification of molecular subtypes and discovery of new pathways via phosphoproteomics [194]. The novel concept of using proteogenomics as an area of research at the interface of proteomics and genomics allowed us to understand the molecular complexity of cancer processes starting from the genome all the way into aberrant translational products. A particular challenging population that may benefit greatly from precision oncology, promoting progression towards the road of personalized medicine, is HIV-infected patients [195,196]. Their predisposition to developing resistant and aggressive malignancies towards treatment when compared to non-HIV-infected patients opens the avenue for more detailed studies [120]. Cancer in HIV patients can be classified into two main categories: AIDS-defining, for which the clinical presence is used to diagnose AIDS in an HIV patient, and non-AIDS-defining, which relates to cancer not solely caused by HIV-1 but also the risk of said cancer being increased in HIV patients or behaving more aggressively. The three malignancies that are classified as AIDS-defining cancers are Kaposi’s sarcoma, aggressive B-cell lymphomas (including Burkitt’s’ lymphoma, diffuse large B-cell lymphoma, primary CNS lymphoma, primary effusion lymphoma, Plasmablastic lymphoma), and invasive cervical cancer [120].

Quantitative proteomic approaches coupled to personalized medicine have expanded the possibilities regarding integrating new emerging technologies that can positively impact the health modalities of the individual. Among these, nanotechnology has been shown to synergize with the use of MS-based approaches for disease progression [197]. This is evidenced by using nanomaterials to aid and facilitate sample processing, digestion, and interactions between each proteoform. There has been a growing number of studies focusing on the use of several classifications of micro beads (magnetic, agarose, or chromatographic) approaches to enrich the digestion process of cell lysates [198,199,200,201]. The use of artificial intelligence (AI) has also overlapped with both proteomics and nanotechnologies to evolve strategies to facilitate the care generating data. Recently, this technology has been successfully implemented into automated AI robots instructed to handle delicate experiments involving liquid samples [202]. This approach has shown both high sensitivity and reproducibility rates for FFPE tissues [203] and cell line cultures [204]. Therefore, new technology continues to be integrated among existing models, with the goal of advancing therapeutics treatments in a fast and precise manner. 

In relation to non-AIDS-defining cancers include lung cancer, anal cancer, Hodgkin’s lymphoma, oropharyngeal cancer, hepatocellular carcinoma, vulvar cancer, penile cancer, and glioblastoma multiforme [119,205,206,207]. The use of proteomics has shown promising results in personalized medical cancer treatment via precision oncology and will surely allow for the more accurate and precise screening of the heterogenous nature of cancer down to the genetic and molecular level, instead of being limited to being histologically centered [208]. Regarding said screening, the selection of the more appropriate therapy along with the appropriate dosage will allow us to optimize the benefits vs side effects in patients on a case-by-case basis in terms of susceptibility or resistance [209]. Furthermore, there have been recent advances in immunotherapy and targeted protein degradation therapy, allowing us to target many proteins previously considered undruggable by pharmacotherapy [210,211,212,213]. By combining proteomics data successfully in a clinical setting, new therapies for not just cancer but also other potential diseases may be possible. While precision medicine aims to improve the patient’s wellbeing and despite the recent advancements in this field, there remains a number of significant holdbacks associated with legal, ethical, and social disputes. Among these, cost efficiency is the most common cause of concern due to the manufacturing of precise pharmacological treatments that specifically target the person’s disease [214]. Since proteomic and other multi-omic approaches are based on metadata for genetic, epigenetic, metabolic, and protein information, meticulous strategies must be ensured to protect the patient’s identity. A security breach of personal data from the individual is considered a strong ethical and legal concern and should be an indicator that the future of personalized medicine needs careful consideration before being administered in the pharmaceutical industry [215,216]. The merging of personal data across various platforms or software increases the probability of a breach of data occurring. The introduction of personalized medicines to healthcare providers also meets another issue due to the need to prove if they can outperform traditional medicines [216]. A lack of expertise for this highly sophisticated field is also a problem when handling this process. The manpower to carry out the tasks of organization, keeping track of personal medicine, and managing the algorithm within a company software with minimal to no mistakes needs to be considered in the future [215]. 

## 7. The Critical Role of Bioinformatics

The signaling mechanisms pertaining to cancer progression have challenged novel integration for bioinformatic tools. As a result, cancer proteome databases have been extensively created to monitor and compile massive data for interpretation. This gives meaningful insight into cancer progression. Consequently, bioinformatics remains an indispensable tool for analyzing large-scale datasets in the most common form of protein and gene expressions. This has led to databases relating to amino acid sequences for a detailed study of the protein structures and mechanistic signaling components in cancer. In recent years, the advancements in bioinformatics have significantly increased as an integral tool for supporting therapeutic cancer treatments. Identifying new classes of molecular signatures such as alternative splicing reflects one of these advancements, which was previously unattainable [217]. Newly synthesized drugs that are currently being tested for distinct classifications of oncological diseases are also the results of these novel intuitive methods for bioinformatical approaches [218]. As these tools continue to evolve along with robust and established databases such as the Cancer Genome Atlas (TCGA) and Gene Expression Omnibus (GEO), the use of quantitative proteomics has become an acceptable practice. An example for this would be the sharing of user-generated proteomic data from researchers that can provide a comprehensive and insightful advancement towards the understanding of the molecular mechanism in place. In a recent study conducted in PCa, bioinformatics was implemented with the aim of screening and cataloguing crucial genes that regulate biological processes of interest, with insightful views relating to tumorigenesis and future therapeutic implementation [219]. A model of this application is the use of gene chips to screen for significant biomarkers that lead towards disease pathogenesis, as conducted in a recent TNBC study [220]. The incorporation of gene chips with quantitative high-throughput proteomics techniques allows for a powerful and efficient gene sequencing tool for mass-scale analyzing differentially regulated genes between normal and tumorigenic tissues. In a similar manner, from previous examples, the integration of cancer bioinformatics has led to the recent discovery of ribosomal oxygenase 2 (RIOX2) as a key oncogene in lung cancer, which also participates as a common factor in a variety of carcinogenic disturbances [221]. In another study correlating with lung cancer, bioinformatic analysis was used to identify unique proteomic profiles of patient subtypes associated with sex, response, and tumor progression [222]. Moreover, selected database classifications do not detect sub-proteomes levels of brain, extracellular vesicles, and cancer-related proteins in GBM [223]. This review aimed to understand the highly infiltrative abilities of GBM as well as the relapse rate that characterizes its aggressiveness for unexplored datasets. Gene ontology analysis has revealed the over-expressed regulation of blood coagulation and plasminogen that can act as a gateway for continues blood–brain barrier damage. Here, we highlight some of the latest bioinformatics integrations alongside the use of quantitative proteomics for detecting and determining some of the most prominent cancer phenomena. Recent rapid biotechnological breakthroughs with large datasets have set new challenges for the identification of complex and unique molecular features that drive malignancies and the molecular mapping of cancer-related disparities. Therefore, the foundations of bioinformatic continue to evolve with novel applications of computational methods, also forming an integral part of proteomic-based technologies.

## 8. Conclusions

Proteomics profiling is a continuously expanding field relying on the combination of current advances, both technological and analytical, to screen for and discover cancer-causing molecular signatures. These proteomics signatures and signaling including ER stress have been implicated in various cancers and provide, as such, the potential to develop effective therapies and bioinformatic resources for basic and translational research. Moreover, information on large-scale proteomics studies on clinical models with targetable approaches such as metastasis, metabolism, proliferation, cell cycle, and stress-induced responses (Figure 3 and Figure 4) provide insight into tumor development relating to current drug treatments and viral infections. Targeting overlapping pathways can lead to a complete understanding of frequently modulated pathways shared among similar tumor environments. Bioinformatical databases that are specifically created for monitoring general disease progression are well-organized in nature. However, these are still inferior in comparison to cancer-oriented computational databases. A comprehensive mechanistic quantitative proteomics analysis should be integrated in most disease studies to address this issue. In this regard, proteomics can be considered as an integral part of cancer research for identifying potential biomarkers and advanced diagnostics. In this review, we discussed recent mechanistic molecular proteomics signatures and bioinformatical approaches. Studying these specific molecular markers provides a powerful approach for diagnosis and therapeutics in a vast array of cancers (TBNC, lung cancer, PCa, GBM, Rb, and HIV-associated malignancies). The identified protein markers in patients with these metastatic solid tumors can be seen as early mechanistic insights into biological processes and oncogenic disparities. In hindsight, proteomics promises to contribute to personalized medicine by allowing us to comprehensively understand mechanistic molecular signatures as unique tools for biomarker discovery. Such implementations will be reflected in current novel technology trends such as the use of nanomedicine and artificial intelligence (AI). The combination of proteomics with these emerging technologies is likely to play a key role in uncovering the oncological mechanisms for cancer diagnosis, prognosis, and monitoring. Ultimately, proteomics strives to guide targeted therapy, to stratify cancer, and to identify evidence of resistance and redirect treatment strategies much earlier.

## References

[1] Al-Amrani S., Al-Jabri Z., Al-Zaabi A., Alshekaili J., Al-Khabori M. (2021). Proteomics: Concepts and Applications in Human Medicine. World J. Biol. Chem..

[2] Al Shweiki M.R., Oeckl P., Steinacker P., Barschke P., Dorner-Ciossek C., Hengerer B., Schönfeldt-Lecuona C., Otto M. (2020). Proteomic Analysis Reveals a Biosignature of Decreased Synaptic Protein in Cerebrospinal Fluid of Major Depressive Disorder. Transl. Psychiatry.

[3] Hermann J., Schurgers L., Jankowski V. (2022). Identification and Characterization of Post-Translational Modifications: Clinical Implications. Mol. Asp. Med..

[4] Kwon Y.W., Jo H.-S., Bae S., Seo Y., Song P., Song M., Yoon J.H. (2021). Application of Proteomics in Cancer: Recent Trends and Approaches for Biomarkers Discovery. Front. Med..

[5] Yang X.-L., Shi Y., Zhang D.-D., Xin R., Deng J., Wu T.-M., Wang H.-M., Wang P.-Y., Liu J.-B., Li W. (2021). Quantitative Proteomics Characterization of Cancer Biomarkers and Treatment. Mol. Ther. Oncolytics.

[6] Cozzolino F., Landolfi A., Iacobucci I., Monaco V., Caterino M., Celentano S., Zuccato C., Cattaneo E., Monti M. (2020). New Label-Free Methods for Protein Relative Quantification Applied to the Investigation of an Animal Model of Huntington Disease. PLoS ONE.

[7] Saleh A.M., Wilding K.M., Calve S., Bundy B.C., Kinzer-Ursem T.L. (2019). Non-Canonical Amino Acid Labeling in Proteomics and Biotechnology. J. Biol. Eng..

[8] Čuklina J., Lee C.H., Williams E.G., Sajic T., Collins B.C., Rodríguez Martínez M., Sharma V.S., Wendt F., Goetze S., Keele G.R. (2021). Diagnostics and Correction of Batch Effects in Large-Scale Proteomic Studies: A Tutorial. Mol. Syst. Biol..

[9] Miles H.N., Delafield D.G., Li L. (2021). Recent Developments and Applications of Quantitative Proteomics Strategies for High-Throughput Biomolecular Analyses in Cancer Research. RSC Chem. Biol..

[10] Koziol J., Griffin N., Long F., Li Y., Latterich M., Schnitzer J. (2013). On Protein Abundance Distributions in Complex Mixtures. Proteome Sci..

[11] Zhang W., Sakashita S., Taylor P., Tsao M.S., Moran M.F. (2015). Comprehensive Proteome Analysis of Fresh Frozen and Optimal Cutting Temperature (OCT) Embedded Primary Non-Small Cell Lung Carcinoma by LC–MS/MS. Methods.

[12] Dapic I., Uwugiaren N., Jansen P., Corthals G.L. (2017). Fast and Simple Protocols for Mass Spectrometry-Based Proteomics of Small Fresh Frozen Uterine Tissue Sections. Anal. Chem..

[13] Zhao X., Huffman K.E., Fujimoto J., Canales J.R., Girard L., Nie G., Heymach J.V., Wistuba I.I., Minna J.D., Yu Y. (2017). Quantitative Proteomic Analysis of Optimal Cutting Temperature (OCT) Embedded Core-Needle Biopsy of Lung Cancer. J. Am. Soc. Spectrom..

[14] Sprung R.W., Brock J.W.C., Tanksley J.P., Li M., Washington M.K., Slebos R.J.C., Liebler D.C. (2009). Equivalence of Protein Inventories Obtained from Formalin-Fixed Paraffin-Embedded and Frozen Tissue in Multidimensional Liquid Chromatography-Tandem Mass Spectrometry Shotgun Proteomic Analysis. Mol. Cell. Proteom..

[15] Macklin A., Khan S., Kislinger T. (2020). Recent Advances in Mass Spectrometry Based Clinical Proteomics: Applications to Cancer Research. Clin. Proteom..

[16] Liotta L.A., Pappalardo P.A., Carpino A., Haymond A., Howard M., Espina V., Wulfkuhle J., Petricoin E. (2021). Laser Capture Proteomics: Spatial Tissue Molecular Profiling from the Bench to Personalized Medicine. Expert Rev. Proteom..

[17] Alghanem B., Ali R., Nehdi A., Al Zahrani H., Altolayyan A., Shaibah H., Baz O., Alhallaj A., Moresco J.J., Diedrich J.K. (2020). Proteomics Profiling of KAIMRC1 in Comparison to MDA-MB231 and MCF-7. Int. J. Mol. Sci..

[18] Curran S. (2000). Laser Capture Microscopy. Mol. Pathol..

[19] Dapic I., Uwugiaren N., Kers J., Mohammed Y., Goodlett D.R., Corthals G. (2022). Evaluation of Fast and Sensitive Proteome Profiling of FF and FFPE Kidney Patient Tissues. Molecules.

[20] Wimmer I., Tröscher A.R., Brunner F., Rubino S.J., Bien C.G., Weiner H.L., Lassmann H., Bauer J. (2018). Systematic Evaluation of RNA Quality, Microarray Data Reliability and Pathway Analysis in Fresh, Fresh Frozen and Formalin-Fixed Paraffin-Embedded Tissue Samples. Sci. Rep..

[21] Pais R.J., Jardine C., Zmuidinaite R., Lacey J., Butler S., Iles R. (2019). Rapid, Affordable and Efficient Screening of Multiple Blood Abnormalities Made Possible Using an Automated Tool for MALDI-ToF Spectrometry Analysis. Appl. Sci..

[22] Sun H., Poudel S., Vanderwall D., Lee D.-G., Li Y., Peng J. (2022). 29-Plex Tandem Mass Tag Mass Spectrometry Enabling Accurate Quantification by Interference Correction. Proteomics.

[23] Ma H., Li R., Di X., Jin X., Wang Y., Lai B., Shi C., Mingxin J., Zhu X.-R., Wang K. (2019). ITRAQ-Based Proteomic Analysis Reveals Possible Target-Related Proteins in Human Adrenocortical Adenomas. BMC Genom..

[24] Isotope Coded Affinity Tags (ICATTM) (Institute for Systems Biology) | Innovative Molecular Analysis Technologies (IMAT) imat.cancer.gov. https://imat.cancer.gov/about-imat/outputs-and-achievements/individual-technologies-and-platforms/isotope-coded-affinity.

[25] Lottspeich F., Kellermann J. (2011). ICPL Labeling Strategies for Proteome Research. Methods Mol. Biol..

[26] Thompson A., Schäfer J., Kuhn K., Kienle S., Schwarz J., Schmidt G., Neumann T., Hamon C. (2003). Tandem Mass Tags: A Novel Quantification Strategy for Comparative Analysis of Complex Protein Mixtures by MS/MS. Anal. Chem..

[27] Bathla S., Sindhu A., Kumar S., Dubey S.K., Pattnaik S., Rawat P., Chopra A., Dang A., Kaushik J.K., Mohanty A.K. (2020). Tandem Mass Tag (TMT)-Based Quantitative Proteomics Reveals Potential Targets Associated with Onset of Sub-Clinical Mastitis in Cows. Sci. Rep..

[28] Kelstrup C.D., Aizikov K., Batth T.S., Kreutzman A., Grinfeld D., Lange O., Mourad D., Makarov A.A., Olsen J.V. (2018). Limits for Resolving Isobaric Tandem Mass Tag Reporter Ions Using Phase-Constrained Spectrum Deconvolution. J. Proteome Res..

[29] Pottiez G., Wiederin J., Fox H.S., Ciborowski P. (2012). Comparison of 4-Plex to 8-Plex ITRAQ Quantitative Measurements of Proteins in Human Plasma Samples. J. Proteome Res..

[30] Grabowska K., Harwood E., Ciborowski P. (2021). HIV and Proteomics: What We Have Learned from High Throughput Studies. PROTEOMICS Clin. Appl..

[31] Gouw J.W., Krijgsveld J., Heck A.J.R. (2010). Quantitative Proteomics by Metabolic Labeling of Model Organisms. Mol. Cell. Proteom..

[32] Koomen J.M., Haura E.B., Bepler G., Sutphen R., Remily-Wood E.R., Benson K., Hussein M., Hazlehurst L.A., Yeatman T.J., Hildreth L.T. (2008). Proteomic Contributions to Personalized Cancer Care. Mol. Cell. Proteom..

[33] Chen X., Wei S., Ji Y., Guo X., Yang F. (2015). Quantitative Proteomics Using SILAC: Principles, Applications, and Developments. Proteomics.

[34] Chen S.-W., Drechsler R., Gafken P.R., Olsen C.P., Blair (2015). 13C- and 15N-Labeling Strategies Combined with Mass Spectrometry Comprehensively Quantify Phospholipid Dynamics in C. Elegans. PLoS ONE.

[35] Whitelegge J.P., Katz J.E., Pihakari K.A., Hale R., Aguilera R., Gómez S.M., Faull K.F., Vavilin D., Vermaas W. (2004). Subtle Modification of Isotope Ratio Proteomics; an Integrated Strategy for Expression Proteomics. Phytochemistry.

[36] Lau H.-T., Suh H.W., Golkowski M., Ong S.-E. (2014). Comparing SILAC- and Stable Isotope Dimethyl-Labeling Approaches for Quantitative Proteomics. J. Proteome Res..

[37] Luo H., Ge H. (2022). Application of Proteomics in the Discovery of Radiosensitive Cancer Biomarkers. Front. Oncol..

[38] Cox J., Hein M.Y., Luber C.A., Paron I., Nagaraj N., Mann M. (2014). Accurate Proteome-Wide Label-Free Quantification by Delayed Normalization and Maximal Peptide Ratio Extraction, Termed MaxLFQ. Mol. Cell. Proteom..

[39] He B., Shi J., Wang X., Jiang H., Zhu H.-J. (2019). Label-Free Absolute Protein Quantification with Data-Independent Acquisition. J. Proteom..

[40] Rychert J. Benefits and Limitations of MALDI-TOF Mass Spectrometry for the Identification of Microorganisms. https://www.infectiologyjournal.com/articles/benefits-and-limitations-of-malditof-mass-spectrometry-for-the-identification-of-microorganisms.html.

[41] Thermo Fisher Scientific Tandem Mass Tag (TMT) Multiplexing Approach to Protein Quantitation: Q&A. https://www.analyteguru.com/t5/Blog/Tandem-Mass-Tag-TMT-Multiplexing-Approach-to-Protein/ba-p/21253.

[42] Sturm R.M., Lietz C.B., Li L. (2014). Improved Isobaric Tandem Mass Tag Quantification by Ion Mobility Mass Spectrometry. Rapid Commun. Mass Spectrom..

[43] Trinh H.V., Grossmann J., Gehrig P., Roschitzki B., Schlapbach R., Greber U.F., Hemmi S. (2013). ITRAQ-Based and Label-Free Proteomics Approaches for Studies of Human Adenovirus Infections. Int. J. Proteom..

[44] Zhao Y., Lu Q.-Y., Xiao G.G. (2009). Quantitative Proteomics and Biomarker Discovery in Human Cancer. Expert Rev. Proteomics.

[45] Melby J.A., Roberts D.S., Larson E.J., Brown K.A., Bayne E.F., Jin S., Ge Y. (2021). Novel Strategies to Address the Challenges in Top-down Proteomics. J. Am. Soc. Mass Spectrom..

[46] Zhang Y., Fonslow B.R., Shan B., Baek M.-C., Yates J.R. (2013). Protein Analysis by Shotgun/Bottom-up Proteomics. Chem. Rev..

[47] Abolfathi H., Sheikhpour M., Shahraeini S.S., Khatami S., Nojoumi S.A. (2021). Studies in Lung Cancer Cytokine Proteomics: A Review. Expert Rev. Proteom..

[48] Alharbi R.A. (2020). Proteomics Approach and Techniques in Identification of Reliable Biomarkers for Diseases. Saudi J. Biol. Sci..

[49] Boys E.L., Liu J., Robinson P.J., Reddel R.R. (2022). Clinical Applications of Mass Spectrometry-Based Proteomics in Cancer: Where Are We?. Proteomics.

[50] Triple Negative Breast Cancer (Page 6): Pennmedicine.org. https://www.pennmedicine.org/cancer/types-of-cancer/breast-cancer/types-of-breast-cancer/triplenegative-breast-cancer#:~:text=Triple%2Dnegative%20breast%20cancers%20tend.

[51] Asleh K., Negri G.L., Spencer Miko S.E., Colborne S., Hughes C.S., Wang X.Q., Gao D., Gilks C.B., Chia S.K.L., Nielsen T.O. (2022). Proteomic Analysis of Archival Breast Cancer Clinical Specimens Identifies Biological Subtypes with Distinct Survival Outcomes. Nat. Commun..

[52] Cancer Genome Atlas Network (2012). Comprehensive Molecular Portraits of Human Breast Tumours. Nature.

[53] Hoadley K.A., Yau C., Wolf D.M., Cherniack A.D., Tamborero D., Ng S., Leiserson M.D.M., Niu B., McLellan M.D., Uzunangelov V. (2014). Multiplatform Analysis of 12 Cancer Types Reveals Molecular Classification within and across Tissues of Origin. Cell.

[54] Shah S.P., Roth A., Goya R., Oloumi A., Ha G., Zhao Y., Turashvili G., Ding J., Tse K., Haffari G. (2012). The Clonal and Mutational Evolution Spectrum of Primary Triple-Negative Breast Cancers. Nature.

[55] Curtis C., Shah S.P., Chin S.-F., Turashvili G., Rueda O.M., Dunning M.J., Speed D., Lynch A.G., Samarajiwa S., Yuan Y. (2012). The Genomic and Transcriptomic Architecture of 2,000 Breast Tumours Reveals Novel Subgroups. Nature.

[56] Stephens P.J., Tarpey P.S., Davies H., Van Loo P., Greenman C., Wedge D.C., Nik-Zainal S., Martin S., Varela I., Bignell G.R. (2012). The Landscape of Cancer Genes and Mutational Processes in Breast Cancer. Nature.

[57] Krug K., Jaehnig E.J., Satpathy S., Blumenberg L., Karpova A., Anurag M., Miles G., Mertins P., Geffen Y., Tang L.C. (2020). Proteogenomic Landscape of Breast Cancer Tumorigenesis and Targeted Therapy. Cell.

[58] Hughes C.S., McConechy M.K., Cochrane D.R., Nazeran T., Karnezis A.N., Huntsman D.G., Morin G.B. (2016). Quantitative Profiling of Single Formalin Fixed Tumour Sections: Proteomics for Translational Research. Sci. Rep..

[59] Moggridge S., Sorensen P.H., Morin G.B., Hughes C.S. (2018). Extending the Compatibility of the SP3 Paramagnetic Bead Processing Approach for Proteomics. J. Proteome Res..

[60] Anurag M., Zhu M., Huang C., Vasaikar S., Wang J., Hoog J., Burugu S., Gao D., Suman V., Zhang X.H. (2020). Immune Checkpoint Profiles in Luminal B Breast Cancer (Alliance). JNCI J. Natl. Cancer Inst..

[61] Henle A.M., Nassar A., Puglisi-Knutson D., Youssef B., Knutson K.L. (2017). Downregulation of TAP1 and TAP2 in Early Stage Breast Cancer. PLoS ONE.

[62] Liu C., Li Y., Xing X., Zhuang J., Wang J., Wang C., Zhang L., Liu L., Feng F., Li H. (2022). Immunogenomic Landscape Analyses of Immune Molecule Signature-Based Risk Panel for Patients with Triple-Negative Breast Cancer. Mol. Ther. Nucleic. Acids.

[63] Liu R., Li J., Zhang X.Y., Ge X., Ma J. (2023). Differences in Clinical Features and Prognosis between Orbit Adenoid Cystic Carcinoma and Adenocarcinoma: A Study from the SEER 18 Database. Tumori.

[64] Yao Q., Hou W., Chen J., Bai Y., Long M., Huang X., Zhao C., Zhou L., Niu D. (2022). Comparative Proteomic and Clinicopathological Analysis of Breast Adenoid Cystic Carcinoma and Basal-like Triple-Negative Breast Cancer. Front. Med..

[65] Gaur P., Bhattacharya S., Kant S., Kushwaha R.A., Garg R., Singh G., Pandey S. (2019). Association of Inflammatory Biomarkers with Lung Cancer in North Indian Population. Afr. Health Sci..

[66] Enewold L., Mechanic L.E., Bowman E.D., Zheng Y.-L., Yu Z., Trivers G., Alberg A.J., Harris C.C. (2009). Serum Concentrations of Cytokines and Lung Cancer Survival in African Americans and Caucasians. Cancer Epidemiol. Biomark. Prev..

[67] Xu J.Y., Zhang C., Wang X., Zhai L., Ma Y., Mao Y., Qian K., Sun C., Liu Z., Jiang S. (2020). Integrative Proteomic Characterization of Human Lung Adenocarcinoma. Cell.

[68] Herbst R.S., Morgensztern D., Boshoff C. (2018). The biology and management of non-small cell lung cancer. Nature.

[69] Hughes P.E., Caenepeel S., Wu L.C. (2016). Targeted Therapy and Checkpoint Immunotherapy Combinations for the Treatment of Cancer. Trends Immunol..

[70] Lin J.J., Shaw A.T. (2016). Resisting Resistance: Targeted Therapies in Lung Cancer. Trends Cancer.

[71] Peters S., Kerr K.M., Stahel R. (2018). PD-1 blockade in advanced NSCLC: A focus on pembrolizumab. Cancer Treat. Rev..

[72] Thomas A., Liu S.V., Subramaniam D.S., Giaccone G. (2015). Refining the treatment of NSCLC according to histological and molecular subtypes. Nat. Rev. Clin. Oncol..

[73] Hirsch F.R., Varella-Garcia M., Bunn P.A., Di Maria M.V., Veve R., Bremmes R.M., Barón A.E., Zeng C., Franklin W.A. (2003). Epidermal growth factor receptor in non-small-cell lung carcinomas: Correlation between gene copy number and protein expression and impact on prognosis. J. Clin. Oncol..

[74] Vargas A.J., Harris C.C. (2016). Biomarker development in the precision medicine era: Lung cancer as a case study. Nat. Rev. Cancer.

[75] Wang S., Chen A., Zhu W., Feng D., Wei J., Li Q., Shi X., Lv X., Liu M. (2022). Characterization of Fatty Acid Metabolism in Lung Adenocarcinoma. Front. Genet..

[76] Dang C.V., Reddy E.P., Shokat K.M., Soucek L. (2017). Drugging the ‘undruggable’ cancer targets. Nat. Rev. Cancer.

[77] Papke B., Der C.J. (2017). Drugging RAS: Know the enemy. Science.

[78] Key Statistics for Prostate Cancer | Prostate Cancer Facts. https://www.cancer.org/cancer/types/prostate-cancer/about/key-statistics.html.

[79] Screening Tests for Prostate Cancer. https://www.cancer.org/cancer/types/prostate-cancer/detection-diagnosis-staging/tests.html.

[80] Khoo A., Liu L.Y., Nyalwidhe J.O., Semmes O.J., Vesprini D., Downes M.R., Boutros P.C., Liu S.K., Kislinger T. (2021). Proteomic discovery of non-invasive biomarkers of localized prostate cancer using mass spectrometry. Nat. Rev. Urol..

[81] Tonry C., Finn S., Armstrong J., Pennington S.R. (2020). Clinical proteomics for prostate cancer: Understanding prostate cancer pathology and protein biomarkers for improved disease management. Clin. Proteom..

[82] Ummanni R., Duscharla D., Barett C., Venz S., Schlomm T., Heinzer H., Walther R., Bokemeyer C., Brümmendorf T.H., Murthy P.V. (2015). Prostate cancer-associated autoantibodies in serum against tumor-associated antigens as potential new biomarkers. J. Proteom..

[83] Leng S.X., McElhaney J.E., Walston J.D., Xie D., Fedarko N.S., Kuchel G.A. (2008). ELISA and Multiplex Technologies for Cytokine Measurement in Inflammation and Aging Research. J. Gerontol. A Biol. Sci. Med. Sci..

[84] Addona T.A., Shi X., Keshishian H., Mani D.R., Burgess M., Gillette M.A., Clauser K.R., Shen D., Lewis G.D., Farrell L.A. (2011). A Pipeline That Integrates the Discovery and Verification of Plasma Protein Biomarkers Reveals Candidate Markers for Cardiovascular Disease. Nat. Biotechnol..

[85] Reis B.S., Jungbluth A.A., Frosina D., Holz M., Ritter E., Nakayama E., Ishida T., Obata Y., Carver B., Scher H. (2013). Prostate Cancer Progression Correlates with Increased Humoral Immune Response to a Human Endogenous Retrovirus GAG Protein. Clin. Cancer Res..

[86] Kragstrup T.W., Vorup-Jensen T., Deleuran B., Hvid M. (2013). A Simple Set of Validation Steps Identifies and Removes False Results in a Sandwich Enzyme-Linked Immunosorbent Assay Caused by Anti-Animal IgG Antibodies in Plasma from Arthritis Patients. Springerplus.

[87] Sun R., Hunter C., Chen C., Ge W., Morrice N., Liang S., Zhu T., Yuan C., Ruan G., Zhang Q. (2020). Accelerated Protein Biomarker Discovery from FFPE Tissue Samples Using Single-Shot, Short Gradient Microflow SWATH MS. J. Proteome Res..

[88] Randall E.C., Zadra G., Chetta P., Lopez B.G.C., Syamala S., Basu S.S., Agar J.N., Loda M., Tempany C.M., Fennessy F.M. (2019). Molecular Characterization of Prostate Cancer with Associated Gleason Score Using Mass Spectrometry Imaging. Mol. Cancer Res..

[89] Clendinen C.S., Gaul D.A., Monge M.E., Arnold R.S., Edison A.S., Petros J.A., Fernández F.M. (2019). Preoperative Metabolic Signatures of Prostate Cancer Recurrence Following Radical Prostatectomy. J. Proteome Res..

[90] Sadeesh N., Scaravilli M., Latonen L. (2021). Proteomic Landscape of Prostate Cancer: The View Provided by Quantitative Proteomics, Integrative Analyses, and Protein Interactomes. Cancers.

[91] Sinha A., Huang V., Livingstone J., Wang J., Fox N.S., Kurganovs N., Ignatchenko V., Fritsch K., Donmez N., Heisler L.E. (2019). The Proteogenomic Landscape of Curable Prostate Cancer. Cancer Cell.

[92] Latonen L., Afyounian E., Jylhä A., Nättinen J., Aapola U., Annala M., Kivinummi K.K., Tammela T.T.L., Beuerman R.W., Uusitalo H. (2018). Integrative Proteomics in Prostate Cancer Uncovers Robustness against Genomic and Transcriptomic Aberrations during Disease Progression. Nat. Commun..

[93] Customized Drug to Kill Brain Cancer Cells National Institutes of Health (NIH). https://www.nih.gov/news-events/nih-research-matters/customized-drug-kill-brain-cancer-cells#:~:text=A%20type%20of%20tumor%20called.

[94] Chen L., Qin D., Guo X., Wang Q., Li J. (2021). Putting Proteomics into Immunotherapy for Glioblastoma. Front. Immunol..

[95] Lam K.H.B., Leon A.J., Hui W., Lee S.C.-E., Batruch I., Faust K., Klekner A., Hutóczki G., Koritzinsky M., Richer M. (2022). Topographic Mapping of the Glioblastoma Proteome Reveals a Triple-Axis Model of Intra-Tumoral Heterogeneity. Nat. Commun..

[96] Dai X., Shen L. (2022). Advances and Trends in Omics Technology Development. Front. Med..

[97] Jaoude D.A., Moore J.A., Moore M.B., Twumasi-Ankrah P., Ablah E., Moore D.F. (2019). Glioblastoma and Increased Survival with Longer Chemotherapy Duration. Kans. J. Med..

[98] Yi H.-G., Jeong Y.H., Kim Y., Choi Y.-J., Moon H.E., Park S.H., Kang K.S., Bae M., Jang J., Youn H. (2019). A Bioprinted Human-Glioblastoma-On-a-Chip for the Identification of Patient-Specific Responses to Chemoradiotherapy. Nat. Biomed. Eng..

[99] Truong D., Fiorelli R., Barrientos E.S., Melendez E.L., Sanai N., Mehta S., Nikkhah M. (2019). A Three-Dimensional (3D) Organotypic Microfluidic Model for Glioma Stem Cells—Vascular Interactions. Biomaterials.

[100] El Hage S., Wakim E., Daou L., El Masri J., Salameh P. (2021). Epidemiology and Incidence of Retinoblastoma in the Middle East: A Nationwide Study in Lebanon. Cureus.

[101] Hu H., Zhang W., Wang Y., Huang D., Shi J., Li B., Zhang Y., Zhou Y. (2018). Characterization, Treatment and Prognosis of Retinoblastoma with Central Nervous System Metastasis. BMC Ophthalmol..

[102] Wu C., Yang J., Xiao W., Jiang Z., Chen S., Guo D., Zhang P., Liu C., Yang H., Xie Z. (2022). Single-Cell Characterization of Malignant Phenotypes and Microenvironment Alteration in Retinoblastoma. Cell Death Dis..

[103] Mao P., Shen Y., Xu X., Zhong J. (2022). Comprehensive Analysis of the Immune Cell Infiltration Landscape and Immune-Related Methylation in Retinoblastoma. Front. Genet..

[104] Galardi A., Colletti M., Lavarello C., Di Paolo V., Mascio P., Russo I., Cozza R., Romanzo A., Valente P., De Vito R. (2020). Proteomic Profiling of Retinoblastoma-Derived Exosomes Reveals Potential Biomarkers of Vitreous Seeding. Cancers.

[105] Munier F.L. (2014). Classification and management of seeds in retinoblastoma. Ellsworth Lecture Ghent August 24th 2013. Ophthalmic Genet..

[106] Munier F.L., Gaillard M.-C., Balmer A., Soliman S., Podilsky G., Moulin A.P., Beck-Popovic M. (2012). Intravitreal Chemotherapy for Vitreous Disease in Retinoblastoma Revisited: From Prohibition to Conditional Indications. Br. J. Ophthalmol..

[107] “AIDS Related Malignancies” Johns Hopkins Medicine. www.hopkinsmedicine.org/health/conditions-and-diseases/hiv-and-aids/aidsrelated-malignancies.

[108] Isaguliants M., Bayurova E., Avdoshina D., Kondrashova A., Chiodi F., Palefsky J.M. (2021). Oncogenic Effects of HIV-1 Proteins, Mechanisms Behind. Cancers.

[109] Gao Q., Zhu H., Dong L., Shi W., Chen R., Song Z., Huang C., Li J., Dong X., Zhou Y. (2019). Integrated Proteogenomic Characterization of HBV-Related Hepatocellular Carcinoma. Cell.

[110] Mertins P., Mani D.R., Ruggles K.V., Gillette M.A., Clauser K.R., Wang P., Wang X., Qiao J.W., Cao S., Petralia F. (2016). Proteogenomics Connects Somatic Mutations to Signalling in Breast Cancer. Nature.

[111] Tang L., Zeng J., Geng P., Fang C., Wang Y., Sun M., Wang C., Wang J., Yin P., Hu C. (2018). Global Metabolic Profiling Identifies a Pivotal Role of Proline and Hydroxyproline Metabolism in Supporting Hypoxic Response in Hepatocellular Carcinoma. Clin. Cancer Res..

[112] Eckert M.A., Coscia F., Chryplewicz A., Chang J.W., Hernandez K.M., Pan S., Tienda S.M., Nahotko D.A., Li G., Blaženović I. (2019). Proteomics Reveals NNMT as a Master Metabolic Regulator of Cancer-Associated Fibroblasts. Nature.

[113] Bienvenu E., Mukanyangezi M.F., Rulisa S., Martner A., Hasséus B., Vorontsov E., Tobin G., Giglio D. (2021). Changes in the Proteome in the Development of Chronic Human Papillomavirus Infection—A Prospective Study in HIV Positive and HIV Negative Rwandan Women. Cancers.

[114] Pai S.-Y., Lurain K., Yarchoan R. (2021). How Immunodeficiency Can Lead to Malignancy. Hematol. Am. Soc. Hematol. Educ. Program.

[115] Kadiu I., Ricardo-Dukelow M., Ciborowski P., Gendelman H.E. (2007). Cytoskeletal Protein Transformation in HIV-1-Infected Macrophage Giant Cells. J. Immunol..

[116] Haverland N.A., Fox H.S., Ciborowski P. (2014). Quantitative Proteomics by SWATH-MS Reveals Altered Expression of Nucleic Acid Binding and Regulatory Proteins in HIV-1-Infected Macrophages. J. Proteome Res..

[117] Valentín-Guillama G., López S., Kucheryavykh Y., Chorna N., Pérez J., Ortiz-Rivera J., Inyushin M., Makarov V., Valentín-Acevedo A., Quinones-Hinojosa A. (2018). HIV-1 Envelope Protein Gp120 Promotes Proliferation and the Activation of Glycolysis in Glioma Cell. Cancers.

[118] López S.N., Rodríguez-Valentín M., Rivera M., Rodríguez M., Babu M., Cubano L.A., Xiong H., Wang G., Kucheryavykh L., Boukli N.M. (2017). HIV-1 Gp120 Clade B/c Induces a GRP78 Driven Cytoprotective Mechanism in Astrocytoma. Oncotarget.

[119] Yarchoan R., Uldrick T.S. (2018). HIV-Associated Cancers and Related Diseases. N. Engl. J. Med..

[120] National Cancer Institute HIV Infection and Cancer Risk. https://www.cancer.gov/about-cancer/causes-prevention/risk/infectious-agents/hiv-fact-sheet.

[121] Hernández-Ramírez R.U., Shiels M.S., Dubrow R., Engels E.A. (2017). Cancer Risk in HIV-Infected People in the USA from 1996 to 2012: A Population-Based, Registry-Linkage Study. Lancet HIV.

[122] Choy W., Lagman C., Lee S.J., Bui T.T., Safaee M., Yang I. (2016). Impact of Human Immunodeficiency Virus in the Pathogenesis and Outcome of Patients with Glioblastoma Multiforme. Brain Tumor Res. Treat..

[123] Jokonya L., Musara A., Esene I.N., Kabulo K.D.M., Kabeya C.M., Kalangu K.K.N. (2018). Prevalence of Human Immunodeficiency Virus Infection in Brain Glioma Patients: Is the Virus Protective from Gliomas?. Surg. Neurol. Int..

[124] Oliveira V.C.M.D., Gomes T., Ferreira L.C.L., Damian M.M., Silva V.M.F.Q., Araújo J.R., Safe I.P., Ramasawmy R. (2014). Glioblastoma Multiforme in an HIV-Infected Patient: An Unexpected Diagnosis. J. Int. Assoc. Provid. AIDS Care (JIAPAC).

[125] Grande F., Occhiuzzi M., Rizzuti B., Ioele G., De Luca M., Tucci P., Svicher V., Aquaro S., Garofalo A. (2019). CCR5/CXCR4 Dual Antagonism for the Improvement of HIV Infection Therapy. Molecules.

[126] Alkhatib G. (2009). The Biology of CCR5 and CXCR4. Curr. Opin. HIV AIDS.

[127] Ullah T.R. (2019). The Role of CXCR4 in Multiple Myeloma: Cells’ Journey from Bone Marrow to Beyond. J. Bone Oncol..

[128] Anitha A.K., Narayanan P., Ajayakumar N., Sivakumar K.C., Kumar K.S. (2022). Novel Small Synthetic HIV-1 v3 Crown Variants: CCR5 Targeting Ligands. J. Biochem..

[129] Burger M., Glodek A., Hartmann T., Schmitt-Gräff A., Silberstein L.E., Fujii N., Kipps T.J., Burger J.A. (2003). Functional Expression of CXCR4 (CD184) on Small-Cell Lung Cancer Cells Mediates Migration, Integrin Activation, and Adhesion to Stromal Cells. Oncogene.

[130] Kijima T., Maulik G., Ma P.C., Tibaldi E.V., Turner R.E., Rollins B., Sattler M., Johnson B.E., Salgia R. (2002). Regulation of Cellular Proliferation, Cytoskeletal Function, and Signal Transduction through CXCR4 and C-Kit in Small Cell Lung Cancer Cells. Cancer Res..

[131] Choi W.-T., Yang Y., Xu Y., An J. (2014). Targeting Chemokine Receptor CXCR4 for Treatment of HIV-1 Infection, Tumor Progression, and Metastasis. Curr. Top. Med. Chem..

[132] Li R.-J., Zhao L.-J., Zhan Z.-L., Lü X., Gong L.-L., Wang P. (2012). Significance of Expression of Chemokine Receptor and Matrix Metalloproteinase in Small Cell Lung Cancer. Zhonghua Yi Xue Za Zhi.

[133] Akashi T., Koizumi K., Tsuneyama K., Saiki I., Takano Y., Fuse H. (2008). Chemokine Receptor CXCR4 Expression and Prognosis in Patients with Metastatic Prostate Cancer. Cancer Sci..

[134] Debnath B., Xu S., Grande F., Garofalo A., Neamati N. (2013). Small Molecule Inhibitors of CXCR4. Theranostics.

[135] Walenkamp A.M.E., Lapa C., Herrmann K., Wester H.-J. (2017). CXCR4 Ligands: The next Big Hit?. J. Nucl. Med..

[136] Sicoli D., Jiao X., Ju X., Velasco-Velazquez M., Ertel A., Addya S., Li Z., Ando S., Fatatis A., Paudyal B. (2014). CCR5 Receptor Antagonists Block Metastasis to Bone of V-Src-Oncogene-Transformed Metastatic Prostate Cancer Cell Lines. Cancer Res..

[137] Jiao X., Nawab O., Patel T., Kossenkov A.V., Halama N., Jaeger D., Pestell R.G. (2019). Recent Advances Targeting CCR5 for Cancer and Its Role in Immuno-Oncology. Cancer Res..

[138] Hsu S.-K., Chiu C.-C., Dahms H.-U., Chou C.-K., Cheng C.-M., Chang W.-T., Cheng K.-C., Wang H.-M.D., Lin I.L. (2019). Unfolded Protein Response (UPR) in Survival, Dormancy, Immunosuppression, Metastasis, and Treatments of Cancer Cells. Int. J. Mol. Sci..

[139] Chonghaile T.N., Gupta S., John M., Szegezdi E., Logue S.E., Samali A. (2015). BCL-2 Modulates the Unfolded Protein Response by Enhancing Splicing of X-Box Binding Protein-1. Biochem. Biophys. Res. Commun..

[140] Hatok J., Racay P. (2016). Bcl-2 Family Proteins: Master Regulators of Cell Survival. Biomol. Concepts.

[141] Liu C., Jin Y., Fan Z. (2021). The Mechanism of Warburg Effect-Induced Chemoresistance in Cancer. Front. Oncol..

[142] Papadopoli D., Pollak M., Topisirovic I. (2021). The Role of GSK3 in Metabolic Pathway Perturbations in Cancer. Biochim. Biophys. Acta Mol. Cell Res..

[143] Shah A., Kumar S., Simon S.D., Singh D.P., Kumar A. (2013). HIV Gp120- and Methamphetamine-Mediated Oxidative Stress Induces Astrocyte Apoptosis via Cytochrome P450 2E1. Cell Death Dis..

[144] Ivanov A.V., Valuev-Elliston V.T., Ivanova O.N., Kochetkov S.N., Starodubova E.S., Bartosch B., Isaguliants M.G. (2016). Oxidative Stress during HIV Infection: Mechanisms and Consequences. Oxid. Med. Cell. Longev..

[145] Bayurova E., Jansons J., Skrastina D., Smirnova O., Mezale D., Kostyusheva A., Kostyushev D., Petkov S., Podschwadt P., Valuev-Elliston V. (2019). HIV-1 Reverse Transcriptase Promotes Tumor Growth and Metastasis Formation via ROS-Dependent Upregulation of Twist. Oxid. Med. Cell. Longev..

[146] Miller I.P., Pavlović I., Poljšak B., Šuput D., Milisav I. (2019). Beneficial Role of ROS in Cell Survival: Moderate Increases in H_2_O_2_ Production Induced by Hepatocyte Isolation Mediate Stress Adaptation and Enhanced Survival. Antioxidants.

[147] Yip H.Y.K., Papa A. (2021). Signaling Pathways in Cancer: Therapeutic Targets, Combinatorial Treatments, and New Developments. Cells.

[148] Huang J., Pan H., Wang J., Wang T., Huo X., Ma Y., Lu Z., Sun B., Jiang H. (2021). Unfolded Protein Response in Colorectal Cancer. Cell Biosci..

[149] Khaled J., Kopsida M., Lennernäs H., Heindryckx F. (2022). Drug Resistance and Endoplasmic Reticulum Stress in Hepatocellular Carcinoma. Cells.

[150] Guan R., Zhang X., Guo M. (2020). Glioblastoma Stem Cells and Wnt Signaling Pathway: Molecular Mechanisms and Therapeutic Targets. Chin. Neurosurg. J..

[151] Latour M., Her N.-G., Kesari S., Nurmemmedov E. (2021). WNT Signaling as a Therapeutic Target for Glioblastoma. Int. J. Mol. Sci..

[152] Xu X., Zhang M., Xu F., Jiang S. (2020). Wnt Signaling in Breast Cancer: Biological Mechanisms, Challenges and Opportunities. Mol. Cancer.

[153] Abreu de Oliveira W.A., El Laithy Y., Bruna A., Annibali D., Lluis F. (2022). Wnt Signaling in the Breast: From Development to Disease. Front. Cell Dev. Biol..

[154] Ma J., Qin L., Li X. (2020). Role of STAT3 Signaling Pathway in Breast Cancer. Cell Commun. Signal..

[155] Wang W., Nag S., Zhang R. (2015). Targeting the NFκB Signaling Pathways for Breast Cancer Prevention and Therapy. Curr. Med. Chem..

[156] BeLow M., Osipo C. (2020). Notch Signaling in Breast Cancer: A Role in Drug Resistance. Cells.

[157] Shorning B.Y., Dass M.S., Smalley M.J., Pearson H.B. (2020). The PI3K-AKT-MTOR Pathway and Prostate Cancer: At the Crossroads of AR, MAPK, and WNT Signaling. Int. J. Mol. Sci..

[158] Wang C., Chen Q., Xu H. (2021). Wnt/β-Catenin Signal Transduction Pathway in Prostate Cancer and Associated Drug Resistance. Discov. Oncol..

[159] Brighi N., Conteduca V., Lolli C., Gurioli G., Schepisi G., Palleschi M., Mariotti M., Casadei C., De Giorgi U. (2021). The Cyclin-Dependent Kinases Pathway as a Target for Prostate Cancer Treatment: Rationale and Future Perspectives. Crit. Rev. Oncol. Hematol..

[160] Siddiqui F., Vaqar S., Siddiqui A.H. (2022). Lung Cancer.

[161] Sudhesh Dev S., Zainal Abidin S.A., Farghadani R., Othman I., Naidu R. (2021). Receptor Tyrosine Kinases and Their Signaling Pathways as Therapeutic Targets of Curcumin in Cancer. Front. Pharmacol..

[162] Saigí M., Carcereny E., Morán T., Cucurull M., Domènech M., Hernandez A., Martinez-Cardús A., Pros E., Sanchez-Cespedes M. (2022). Biological and Clinical Perspectives of the Actionable Gene Fusions and Amplifications Involving Tyrosine Kinase Receptors in Lung Cancer. Cancer Treat. Rev..

[163] Makino Y., Arakawa Y., Yoshioka E., Shofuda T., Minamiguchi S., Kawauchi T., Tanji M., Kanematsu D., Nonaka M., Okita Y. (2021). Infrequent RAS Mutation Is Not Associated with Specific Histological Phenotype in Gliomas. BMC Cancer.

[164] Kciuk M., Gielecińska A., Budzinska A., Mojzych M., Kontek R. (2022). Metastasis and MAPK Pathways. Int. J. Mol. Sci..

[165] Yang J., Nie J., Ma X., Wei Y., Peng Y., Wei X. (2019). Targeting PI3K in Cancer: Mechanisms and Advances in Clinical Trials. Mol. Cancer.

[166] Colardo M., Segatto M., Di Bartolomeo S. (2021). Targeting RTK-PI3K-MTOR Axis in Gliomas: An Update. Int. J. Mol. Sci..

[167] Read A., Schröder M. (2021). The Unfolded Protein Response: An Overview. Biology.

[168] Bhattarai K.R., Chaudhary M., Kim H.-R., Chae H.-J. (2020). Endoplasmic Reticulum (ER) Stress Response Failure in Diseases. Trends Cell Biol..

[169] Choi J.-A., Song C.-H. (2020). Insights into the Role of Endoplasmic Reticulum Stress in Infectious Diseases. Front. Immunol..

[170] Goda J., Pachpor T., Basu T., Chopra S., Gota V. (2016). Targeting the AKT Pathway: Repositioning HIV Protease Inhibitors as Radiosensitizers. Indian J. Med. Res..

[171] Gupta A.K., Cerniglia G.J., Mick R., McKenna W.G., Muschel R.J. (2005). HIV Protease Inhibitors Block Akt Signaling and Radiosensitize Tumor Cells Both in Vitro and *in Vivo*. Cancer Res..

[172] Wu Y. (2009). The Co-Receptor Signaling Model of HIV-1 Pathogenesis in Peripheral CD4 T Cells. Retrovirology.

[173] Borsa M., Ferreira P.L.C., Petry A., Ferreira L.G.E., Camargo M.M., Bou-Habib D.C., Pinto A.R. (2015). HIV Infection and Antiretroviral Therapy Lead to Unfolded Protein Response Activation. Virol. J..

[174] Liu H., Yang J., Li L., Shi W., Yuan X., Wu L. (2016). The Natural Occurring Compounds Targeting Endoplasmic Reticulum Stress. Evid. Based Complement. Alternat. Med..

[175] Martucciello S., Masullo M., Cerulli A., Piacente S. (2020). Natural Products Targeting ER Stress, and the Functional Link to Mitochondria. Int. J. Mol. Sci..

[176] Kim C., Kim B. (2018). Anti-Cancer Natural Products and Their Bioactive Compounds Inducing ER Stress-Mediated Apoptosis: A Review. Nutrients.

[177] Liu S., Ou S., Huang H. (2017). Green Tea Polyphenols Induce Cell Death in Breast Cancer MCF-7 Cells through Induction of Cell Cycle Arrest and Mitochondrial-Mediated Apoptosis. J. Zhejiang Univ. Sci. B.

[178] Fatima I., Kanwal S., Mahmood T. (2019). Natural Products Mediated Targeting of Virally Infected Cancer. Dose Response.

[179] Rivera M., Ramos Y., Rodríguez-Valentín M., López-Acevedo S., Cubano L.A., Zou J., Zhang Q., Wang G., Boukli N.M. (2017). Targeting Multiple Pro-Apoptotic Signaling Pathways with Curcumin in Prostate Cancer Cells. PLoS ONE.

[180] Burton L.J., Rivera M., Hawsawi O., Zou J., Hudson T., Wang G., Zhang Q., Cubano L., Boukli N., Odero-Marah V. (2016). Muscadine Grape Skin Extract Induces an Unfolded Protein Response-Mediated Autophagy in Prostate Cancer Cells: A TMT-Based Quantitative Proteomic Analysis. PLoS ONE.

[181] Lin C.-L., Lee C.-H., Chen C.-M., Cheng C.-W., Chen P.-N., Ying T.-H., Hsieh Y.-H. (2018). Protodioscin Induces Apoptosis through ROS-Mediated Endoplasmic Reticulum Stress via the JNK/P38 Activation Pathways in Human Cervical Cancer Cells. Cell. Physiol. Biochem..

[182] Yu C.-L., Lee H.-L., Yang S.-F., Wang S.-W., Lin C.-P., Hsieh Y.-H., Chiou H.-L. (2022). Protodioscin Induces Mitochondrial Apoptosis of Human Hepatocellular Carcinoma Cells through Eliciting ER Stress-Mediated IP3R Targeting Mfn1/Bak Expression. J. Hepatocell. Carcinoma.

[183] Lewinska A., Adamczyk-Grochala J., Deregowska A., Wnuk M. (2017). Sulforaphane-Induced Cell Cycle Arrest and Senescence Are Accompanied by DNA Hypomethylation and Changes in MicroRNA Profile in Breast Cancer Cells. Theranostics.

[184] Zou X., Qu Z., Fang Y., Shi X., Ji Y. (2017). Endoplasmic Reticulum Stress Mediates Sulforaphane-Induced Apoptosis of HepG2 Human Hepatocellular Carcinoma Cells. Mol. Med. Rep..

[185] Piotrowski J., Jędrzejewski T., Kozak W. (2015). Immunomodulatory and Antitumor Properties of Polysaccharide Peptide (PSP). Postepy. Hig. Med. Dosw..

[186] Saleh M.H., Rashedi I., Keating A. (2017). Immunomodulatory Properties of Coriolus Versicolor: The Role of Polysaccharopeptide. Front. Immunol..

[187] Rodríguez-Valentín M., López S., Rivera M., Ríos-Olivares E., Cubano L., Boukli N.M. (2018). Naturally Derived Anti-HIV Polysaccharide Peptide (PSP) Triggers a Toll-like Receptor 4-Dependent Antiviral Immune Response. J. Immunol. Res..

[188] Alvarez-Rivera E., Rodríguez-Valentín M., Boukli N.M. (2023). The Antiviral Compound PSP Inhibits HIV-1 Entry via PKR-Dependent Activation in Monocytic Cells. Viruses.

[189] Tang M., Bolderson E., O’Byrne K.J., Richard D.J. (2021). Tumor Hypoxia Drives Genomic Instability. Front. Cell Dev. Biol..

[190] Vadakekolathu J., Boocock D.J., Pandey K., Guinn B., Legrand A., Miles A.K., Coveney C., Ayala R., Purcell A.W., McArdle S.E. (2022). Multi-Omic Analysis of Two Common P53 Mutations: Proteins Regulated by Mutated P53 as Potential Targets for Immunotherapy. Cancers.

[191] Alvarado-Ortiz E., de la Cruz-López K.G., Becerril-Rico J., Sarabia-Sánchez M.A., Ortiz-Sánchez E., García-Carrancá A. (2021). Mutant P53 Gain-of-Function: Role in Cancer Development, Progression, and Therapeutic Approaches. Front. Cell Dev. Biol..

[192] Zhang C., Liu J., Xu D., Zhang T., Hu W., Feng Z. (2020). Gain-of-Function Mutant P53 in Cancer Progression and Therapy. J. Mol. Cell Biol..

[193] Doll S., Gnad F., Mann M. (2019). The Case for Proteomics and Phospho-Proteomics in Personalized Cancer Medicine. Proteom. Clin. Appl..

[194] Rodriguez H., Zenklusen J.C., Staudt L.M., Doroshow J.H., Lowy D.R. (2021). The next Horizon in Precision Oncology—Proteogenomics to Inform Cancer Diagnosis and Treatment. Cell.

[195] Kumbale C.M., Voit E.O. (2021). Toward Personalized Medicine for HIV/AIDS. J. AIDS HIV Treat..

[196] Mu Y., Kodidela S., Wang Y., Kumar S., Cory T.J. (2018). The Dawn of Precision Medicine in HIV: State of the Art of Pharmacotherapy. Expert Opin. Pharmacother..

[197] Zhang Y., Yang H., Yu Y., Zhang Y. (2022). Application of Nanomaterials in Proteomics-Driven Precision Medicine. Theranostics.

[198] Turriziani B., Garcia-Munoz A., Pilkington R., Raso C., Kolch W., von Kriegsheim A. (2014). On-Beads Digestion in Conjunction with Data-Dependent Mass Spectrometry: A Shortcut to Quantitative and Dynamic Interaction Proteomics. Biology.

[199] Hughes C.S., Moggridge S., Müller T., Sorensen P.H., Morin G.B., Krijgsveld J. (2019). Single-Pot, Solid-Phase-Enhanced Sample Preparation for Proteomics Experiments. Nat. Protoc..

[200] Serrels A., Lund T., Serrels B., Byron A., McPherson R.C., von Kriegsheim A., Gómez-Cuadrado L., Canel M., Muir M., Ring J.E. (2015). Nuclear FAK Controls Chemokine Transcription, Tregs, and Evasion of Anti-Tumor Immunity. Cell.

[201] Zhou H., Wang F., Wang Y., Ning Z., Hou W., Wright T.G., Sundaram M., Zhong S., Yao Z., Figeys D. (2011). Improved Recovery and Identification of Membrane Proteins from Rat Hepatic Cells Using a Centrifugal Proteomic Reactor. Mol. Cell. Proteom..

[202] Müller T., Kalxdorf M., Longuespée R., Kazdal D.N., Stenzinger A., Krijgsveld J. (2020). Automated Sample Preparation with SP 3 for Low-Input Clinical Proteomics. Mol. Syst. Biol..

[203] Friedrich C., Schallenberg S., Kirchner M., Ziehm M., Niquet S., Haji M., Beier C., Neudecker J., Klauschen F., Mertins P. (2021). Comprehensive Micro-Scaled Proteome and Phosphoproteome Characterization of Archived Retrospective Cancer Repositories. Nat. Commun.

[204] Ruprecht B., Di Bernardo J., Wang Z., Mo X., Ursu O., Christopher M., Fernandez R.B., Zheng L., Dill B.D., Wang H. (2020). A Mass Spectrometry-Based Proteome Map of Drug Action in Lung Cancer Cell Lines. Nat. Chem. Biol..

[205] Atallah-Yunes S.A., Murphy D.J., Noy A. (2020). HIV-Associated Burkitt Lymphoma. Lancet Haematol..

[206] Clifford D.B., Ances B.M. (2013). HIV-Associated Neurocognitive Disorder. Lancet Infect. Dis..

[207] Eggers C., Arendt G., Hahn K., Husstedt I.W., Maschke M., Neuen-Jacob E., Obermann M., Rosenkranz T., Schielke E., Straube E. (2017). HIV-1-Associated Neurocognitive Disorder: Epidemiology, Pathogenesis, Diagnosis, and Treatment. J. Neurol..

[208] Tsimberidou A.M., Fountzilas E., Nikanjam M., Kurzrock R. (2020). Review of Precision Cancer Medicine: Evolution of the Treatment Paradigm. Cancer Treat. Rev..

[209] Sicklick J.K., Kato S., Okamura R., Schwaederle M., Hahn M.E., Williams C.B., De P., Krie A., Piccioni D.E., Miller V.A. (2019). Molecular Profiling of Cancer Patients Enables Personalized Combination Therapy: The I-PREDICT Study. Nat. Med..

[210] Dale B., Cheng M., Park K.-S., Kaniskan H.Ü., Xiong Y., Jin J. (2021). Advancing Targeted Protein Degradation for Cancer Therapy. Nat. Rev. Cancer.

[211] Davis-Marcisak E.F., Deshpande A., Stein-O’Brien G.L., Ho W.J., Laheru D., Jaffee E.M., Fertig E.J., Kagohara L.T. (2021). From Bench to Bedside: Single-Cell Analysis for Cancer Immunotherapy. Cancer Cell.

[212] Schwaederle M., Zhao M., Lee J.J., Lazar V., Leyland-Jones B., Schilsky R.L., Mendelsohn J., Kurzrock R. (2016). Association of Biomarker-Based Treatment Strategies with Response Rates and Progression-Free Survival in Refractory Malignant Neoplasms. JAMA Oncol..

[213] Schapira M., Calabrese M.F., Bullock A.N., Crews C.M. (2019). Targeted Protein Degradation: Expanding the Toolbox. Nat. Rev. Drug Discov..

[214] Kasztura M., Richard A., Bempong N.-E., Loncar D., Flahault A. (2019). Cost-Effectiveness of Precision Medicine: A Scoping Review. Int. J. Public Health.

[215] Naithani N., Atal A.T., Tilak T.V.S.V.G.K., Vasudevan B., Misra P., Sinha S. (2021). Precision Medicine: Uses and Challenges. Med. J. Armed Forces India.

[216] Goetz L.H., Schork N.J. (2018). Personalized Medicine: Motivation, Challenges, and Progress. Fertil. Steril..

[217] Fu Y., Ling Z., Arabnia H., Deng Y. (2020). Current Trend and Development in Bioinformatics Research. BMC Bioinform..

[218] Behl T., Kaur I., Sehgal A., Singh S., Bhatia S., Al-Harrasi A., Zengin G., Babes E.E., Brisc C., Stoicescu M. (2021). Bioinformatics Accelerates the Major Tetrad: A Real Boost for the Pharmaceutical Industry. Int. J. Mol. Sci..

[219] Guo H., Zhang Z., Wang Y., Xue S. (2021). Identification of Crucial Genes and Pathways Associated with Prostate Cancer in Multiple Databases. J. Int. Med. Res..

[220] Ren Y., Deng R., Zhang Q., Li J., Han B., Ye P. (2020). Bioinformatics Analysis of Key Genes in Triple Negative Breast Cancer and Validation of Oncogene PLK1. Ann. Transl. Med..

[221] Sun B., Zhao H. (2021). The Bioinformatics Analysis of RIOX2 Gene in Lung Adenocarcinoma and Squamous Cell Carcinoma. PLoS ONE.

[222] Harel M., Lahav C., Jacob E., Dahan N., Sela I., Elon Y., Raveh Shoval S., Yahalom G., Kamer I., Zer A. (2022). Longitudinal Plasma Proteomic Profiling of Patients with Non-Small Cell Lung Cancer Undergoing Immune Checkpoint Blockade. J. Immunother. Cancer.

[223] Moresi F., Rossetti D.V., Vincenzoni F., Simboli G.A., La Rocca G., Olivi A., Urbani A., Sabatino G., Desiderio C. (2022). Investigating Glioblastoma Multiforme Sub-Proteomes: A Computational Study of CUSA Fluid Proteomic Data. Int. J. Mol. Sci..

